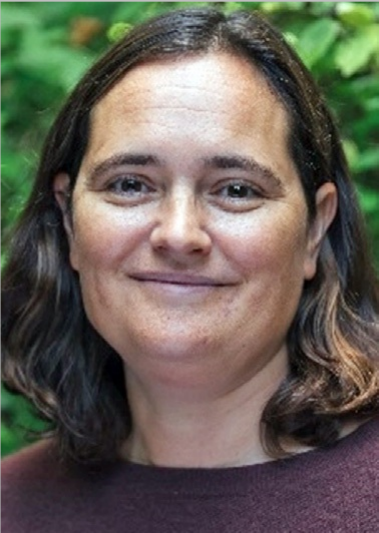# Heterocycle/Heteroallene Ring‐Opening Copolymerization: Selective Catalysis Delivering Alternating Copolymers

**DOI:** 10.1002/anie.202104495

**Published:** 2021-10-19

**Authors:** Alex J. Plajer, Charlotte K. Williams

**Affiliations:** ^1^ Oxford Chemistry Chemical Research Laboratory 12 Mansfield Road Oxford OX1 3TA UK

**Keywords:** epoxides, heteroallenes, heteroatom polymers, polymers, ring-opening copolymerization

## Abstract

Heteroatom‐containing polymers have strong potential as sustainable replacements for petrochemicals, show controllable monomer–polymer equilibria and properties spanning plastics, elastomers, fibres, resins, foams, coatings, adhesives, and self‐assembled nanostructures. Their current and future applications span packaging, house‐hold goods, clothing, automotive components, electronics, optical materials, sensors, and medical products. An interesting route to these polymers is the catalysed ring‐opening copolymerisation (ROCOP) of heterocycles and heteroallenes. It is a living polymerization, occurs with high atom economy, and creates precise, new polymer structures inaccessible by traditional methods. In the last decade there has been a renaissance in research and increasing examples of commercial products made using ROCOP. It is better known in the production of polycarbonates and polyesters, but is also a powerful route to make N‐, S‐, and other heteroatom‐containing polymers, including polyamides, polycarbamates, and polythioesters. This Review presents an overview of the different catalysts, monomer combinations, and polymer classes that can be accessed by heterocycle/heteroallene ROCOP.

## Graphical abstract

Ring‐opening copolymerisation (ROCOP) is a powerful living polymerisation that yields numerous alternating copolymers with precisely placed heteroatoms in the polymer backbones. This Review summarizes the state‐of‐the‐art of this important method, describes the principles of the catalysis, and illustrates the potential of the different monomer combinations and resulting polymers. Trends and future challenges of this approach and the resulting materials are highlighted.

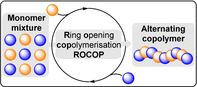

## 1 Introduction

Synthetic polymers are structurally tuneable materials integral to modern life, whether for commodity products, such as clothing, food packaging, or house‐hold goods, or for specialist applications, such as microelectronics, renewable energy generation, or robotics.^[1]^ With worldwide production volumes exceeding 370 M tonnes annually, a polymer‐free society is at best a vague memory rather than a vision for the future.^[2]^ The success story of polymers from last century has its origin in their close coupling with the liquid fuel industry, optimized production methods, low costs, and immense chemical diversity; these features allow material properties to be precisely tailored to a huge range of different applications. In the coming century, a move away from petrochemical raw materials allows a more widespread consideration of chemistry beyond hydrocarbons. There should be advantages to such chemical approaches which are closer to monomer–polymer equilibria and hence should facilitate complete depolymerisation, chemical recycling, and even bio‐degradation.[^2^, ^3^, ^4^] Heteroatom‐containing polymers are experiencing a renaissance, both because of their potential to address such sustainability priorities as well as to deliver new or better properties that target future applications. Currently, sulfur cross‐linked polymers are essential as rubbers and engineering thermoplastics, while amide linkages are, of course, integral to the performances of nylon fibres and urethanes as well as PU foams.[^1^, ^5^, ^6^, ^7^] Most of these materials are made by versatile and scalable polycondensations. Future economic, environment, and technological challenges may benefit from materials better tailored to the application.

**Figure 1:**
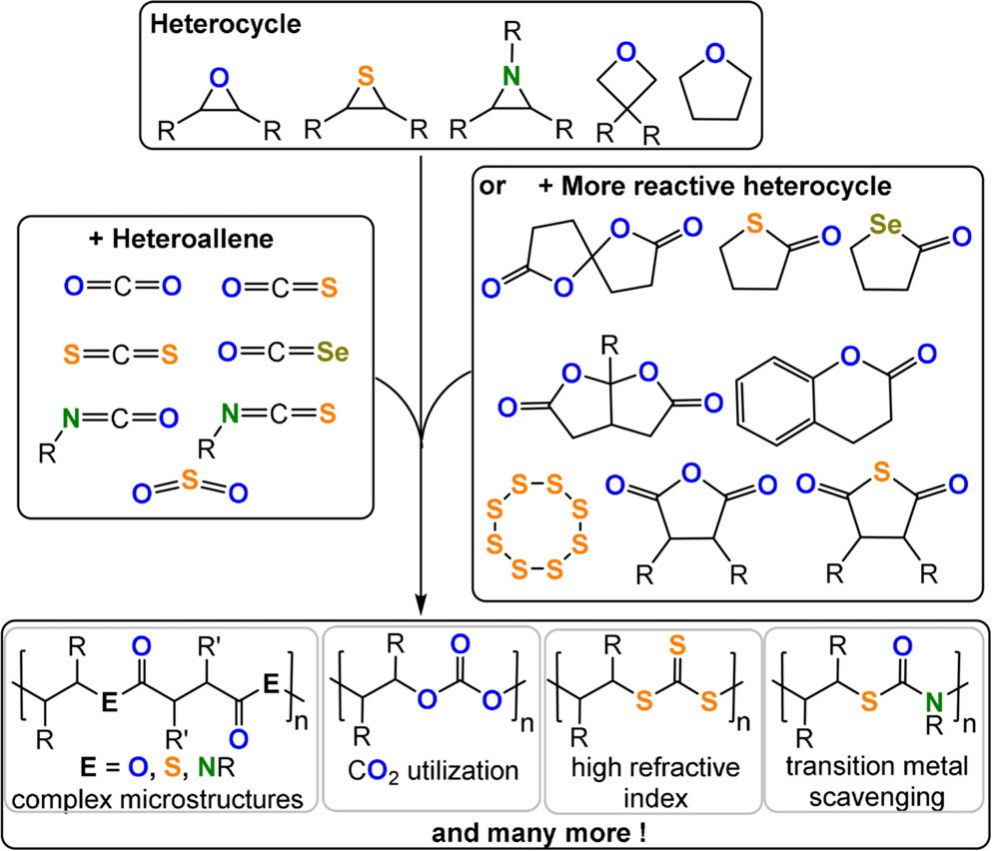
Summary of ROCOP monomer combinations presented in this Review.

One option is to improve structural control and hence provide insight into structure–property relationships of features such as heteroatom placement, functionalised block polymer sequence, architecture, molar mass value, and distribution as well as regio‐ and stereochemistry. Delivering such tunability requires controlled polymerisation methods and one interesting and generally applicable option to install heteroatoms is heterocycle/heteroallene ring‐opening copolymerisation (ROCOP; Figure 1).[^8^, ^9^] Controlled, or living, polymerization shows complete and fast chain initiation followed by uniform propagation rates, limited side reactions, and triggered termination. The resulting polymers show predictable molar mass values, narrow dispersities, compositions dictated by the starting monomer stoichiometry, and high end‐group fidelity; as the growing polymer chain ends are “living” they may be used to make (block) copolymers and more complex architectures with unparalleled selectivity.[^10^, ^11^, ^12^] Heterocycle/heteroallene ROCOP typically affords functional polymers such as (thio)esters, (thio)carbonates, carbamates, or urethanes.

Heterocycle/heteroallene ROCOP dates from the 1960s and renewed attention is partly driven by the potential for sustainability, as monomers such as CO_2_, COS, SO_2_, and S_8_ are industrial wastes and others may be bio‐derived.[^13^, ^14^] Applications of these materials naturally depend upon their polymer chemistry and physics, but low molar mass ROCOP polyols are already showing promise as surfactants, coatings, adhesives, or foams, and higher molar mass polymers are showing promise as high refractive index materials, absorbents, supports, and high‐performance plastics.^[15]^

**Figure 2:**
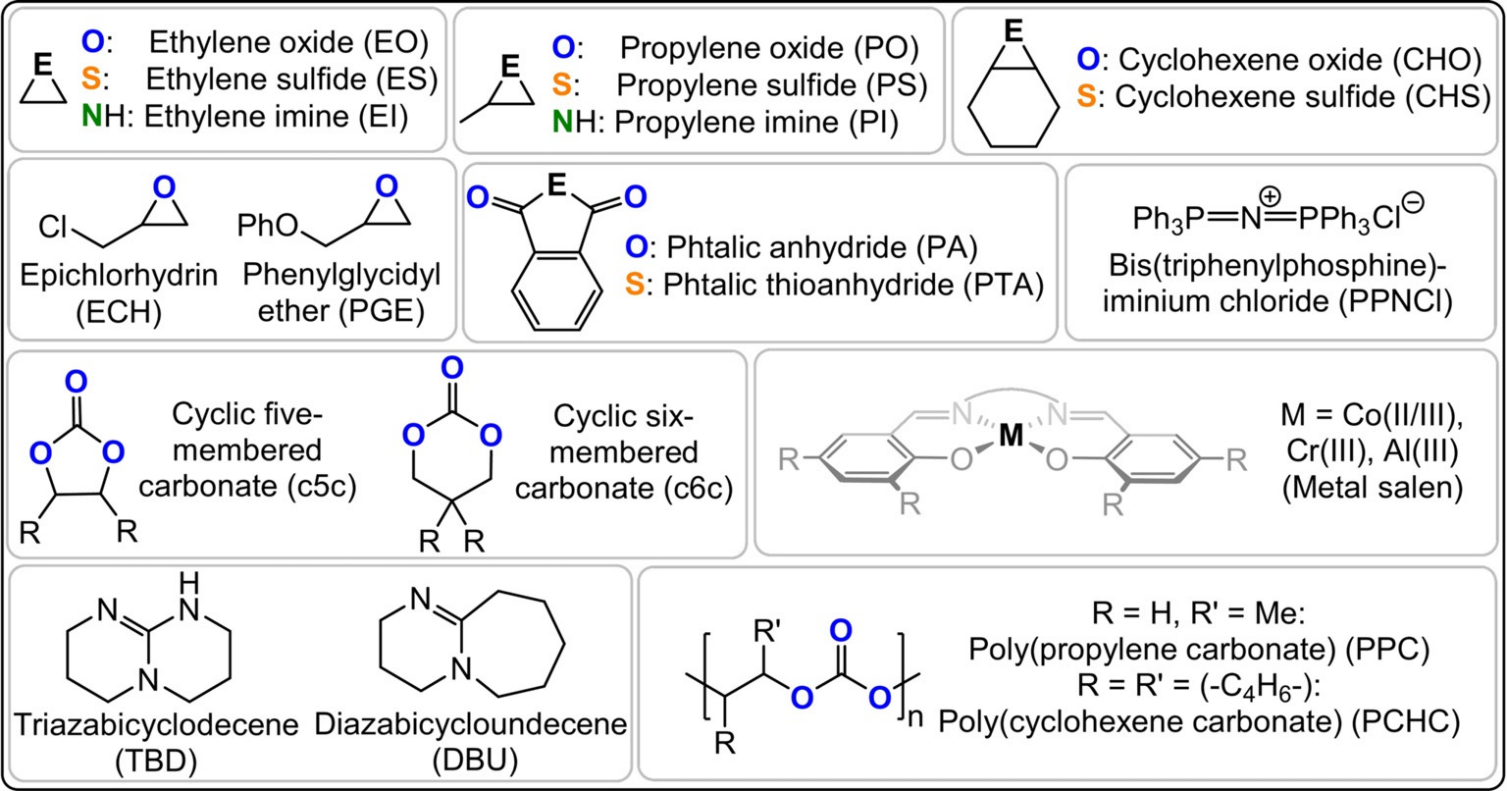
Summary of the structures and acronyms of commonly applied ROCOP monomers.

This Review presents the principles of heterocycle/heteroallene ROCOP catalysis as a tool for polymer synthesis. It introduces the polymerisation methodology, using well‐known monomer combinations such as carbon dioxide/epoxide and anhydride/epoxide ROCOP, and then presents other rarer monomer combinations, with a special focus on polymers containing O, N, or S heteroatoms (Figure 2). Research is progressing fast using such specialized monomer combinations but reproducible, effective, and selective polymer syntheses are essential prior to optimizations of the material properties. In some cases, the activity and selectivity of the ROCOP catalysis is rather low and here areas for future development are highlighted that are driven by promising initial polymer property data. The Review is focussed on polymerisation catalysis, where advances will allow future explorations of polymer properties, processing, and applications.

## 2 Recent Trends in CO_2_/Epoxide ROCOP

Arguably the most widely investigated ROCOP is that of CO_2_ and epoxides. Over the last 50 years this reaction has advanced from a laboratory curiosity to a commercialised technology.[^16^, ^17^] This monomer combination serves as an exemplar of the conceptual advances both in catalysis and product performances. There are already several excellent reviews on CO_2_/epoxide ROCOP and the interested reader is directed to these other reports for comprehensive coverage of this field.[^15^, ^18^, ^19^, ^20^, ^21^, ^22^] The characteristics of CO_2_/epoxide ROCOP provide excellent illustrations of principles that are generally applicable to other, more unusual monomer combinations, and key developments can also inform about future research directions for other polymers.

**Figure 3:**
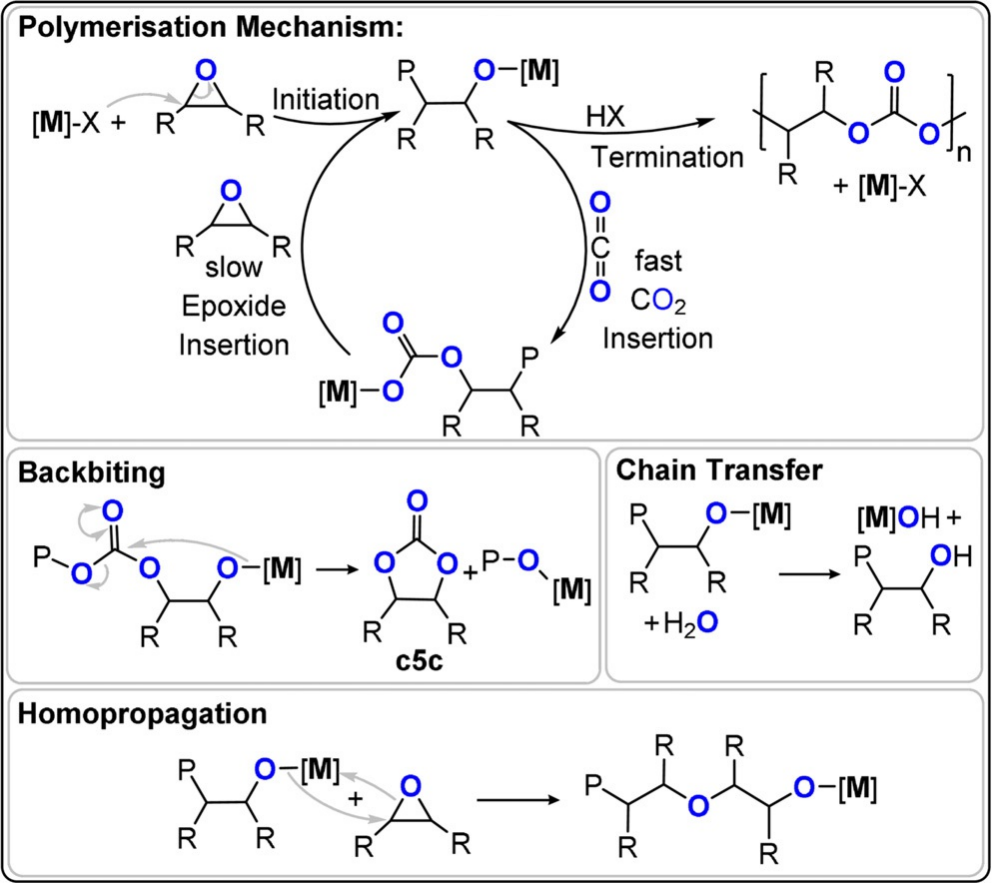
Illustration of the key steps in CO_2_/epoxide ROCOP. [M] refers to a metal catalyst, “P” to the polymer chain, X the initiating co‐ligand.

Firstly, the elementary steps of CO_2_/epoxide ROCOP (Figure 3) are described, since most are general to other heteroallene/heterocycle combinations:

1. Initiation occurs when a coordinated epoxide is ring‐opened, by the initiator/catalyst, to form an alkoxide, which becomes the propagating species. The initiator can be a metal–ligand complex, an ionic co‐catalyst, or a Lewis base.
2. Propagation occurs by two alternating processes: a) CO_2_ insertion, where the alkoxide transforms into a carbonate intermediate. Many catalysts show CO_2_ insertion kinetics that are considerably faster than epoxide ring‐opening. b) Epoxide ring opening, where the carbonate intermediate ring opens the epoxide to regenerate the alkoxide. For many catalysts this is the “rate‐determining step”.
3. Termination occurs when the catalyst is permanently de‐activated, usually by irreversible protonolysis achieved by adding excess water, acid, or even atmospheric moisture into the polymerisation.

Other processes can also occur and these may or may not be desired:

1. Backbiting occurs when the alkoxide or carbonate intermediates attack the polymer chain rather than a new monomer. This process generates “cyclic five‐membered carbonate” (c5c) instead of progressing chain growth. For most carbon dioxide/epoxide coupling reactions, c5c is the thermodynamic product and polycarbonate is the kinetic product.
2. Epoxide homopropagation occurs when the alkoxide intermediate reacts with a second epoxide molecule, rather than with carbon dioxide. It results in the formation of ether, or even polyether, linkages in the polymer chain.
3. Chain transfer occurs when deliberately added protic compounds, typically alcohols, water, or carboxylic acids, undergo a rapid and reversible series of exchange reactions with the propagating alkoxide or carbonate intermediates.^[23]^ Catalysts able to undergo controlled chain‐transfer reactions can be very useful, for example, for precise control over the polymer molar mass or end‐group functionality. These reactions are also exploited to produce multi‐functional star or branched materials. In some contexts, a form of chain transfer is applied to “start” the polymerisations. In other contexts, chain transfer occurs from 1,2‐diols formed by epoxide hydrolysis, which is attributed to low levels of water contamination.[^23^, ^24^] In such cases, the resulting polycarbonates show bimodal molar mass distributions, as a result of chains initiated both by the catalyst and diol.[^25^, ^26^] Some catalysts are very tolerant of chain‐transfer agents, thereby delivering precisely controlled molar mass and directing specific applications.[^27^, ^28^] Low molar mass, hydroxy‐end‐capped polycarbonates are useful as surfactants, polyols, or resin components and for making higher polymers, particularly polyurethanes.^[7]^ Cross‐linking reactions either using end‐ or side‐chain substituents deliver coatings, resins, or thermosets. Higher molar mass polycarbonates are used as elastomers, films, and rigid plastics.^[15]^

**Figure 4:**
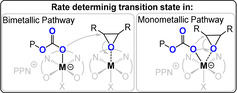
Illustration of the key difference between mono‐ and bimetallic pathways.

Heterocycle/heteroallene ROCOP is critically dependent upon the catalyst selection, and we provide here an outline of the best performing systems. One widely investigated class are metal(III)‐salen or porphyrin catalyst systems which comprise the use of Cr^III^, Co^III^, or Al^III^ complexes together with a nucleophilic co‐catalyst, often a soluble “onium”‐halide salt (consisting of a weakly coordinating cation such as R_4_N^+^, R_4_P^+^, or Ph_3_P=N^+^=PPh_3_ (PPN) and, for example, Cl^−^ or Br^−^) or an organic base (e.g. diazabicycloundecene (DBU) or triazabicyclodecene (TBD)).[^20^, ^29^, ^30^, ^31^, ^32^] These catalyst systems polymerize through bimetallic and/or monometallic mechanisms (Figure 4). Often both mechanisms appear to occur in parallel, differing by epoxide activation at the same or a different metal to the site of carbonate coordination. In either case, catalytic activity is lost or severely compromised without the co‐catalyst and this means that activity also falls rapidly below a critical ion‐pair concentration.^[29]^ At high temperatures, such systems typically deliver large amounts of c5c.^[20]^

**Figure 5:**
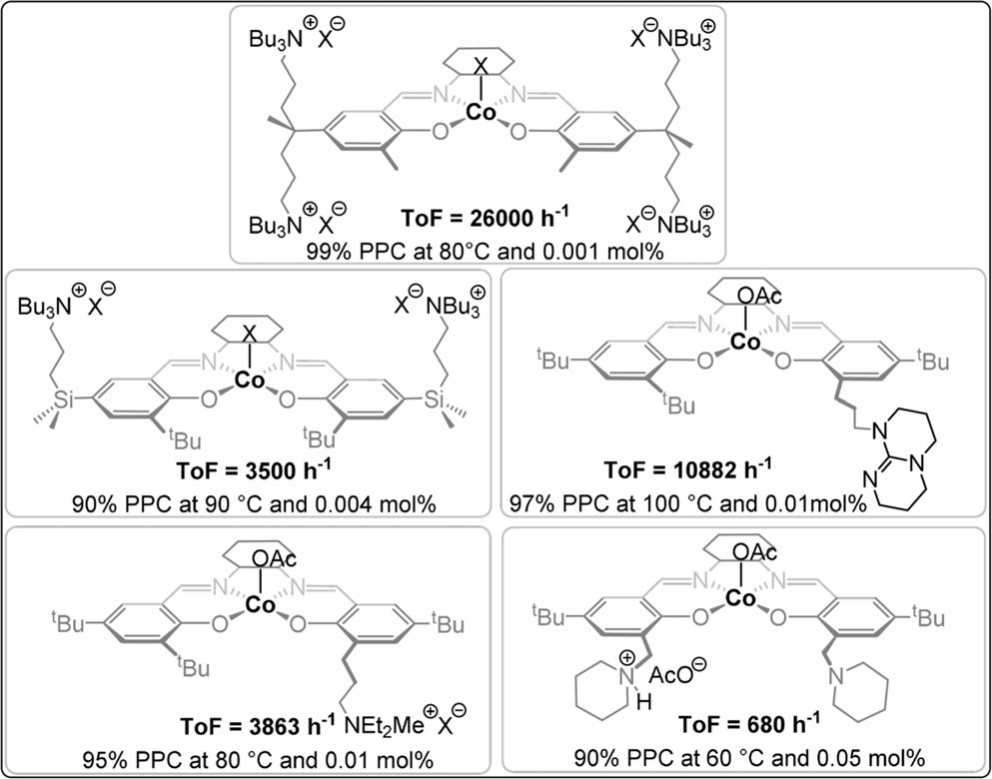
Metal‐salen catalysts with a tethered co‐catalyst; X=2,4‐dinitrophenolate.[^33^, ^34^, ^35^, ^36^]

To address these difficulties, many catalysts were re‐designed to attach the co‐catalyst to the ancillary ligand; this strategy led to outstanding activity values, amongst the highest reported in this field (Figure 5). Nozaki and co‐workers pioneered this strategy, reporting a piperidinium‐tethered Co^III^‐salen complex that showed excellent activity for CO_2_/propene oxide (PO) ROCOP.^[33]^ Later, Lee and co‐workers reported a Co^III^‐salen complex featuring two tethered silylammonium dinitrophenolate co‐catalysts which showed remarkable activity at a low catalyst loading.^[34]^ Even higher rates were achieved by tethering four ammonium(dinitro)phenolate substituents to the Co^III^‐salen complex, although it should be noted the catalyst syntheses were lengthy.^[35]^ Recently, Nozaki and co‐workers reported a four‐arm tethered Al^III^‐porphyrin catalyst that showed excellent activity for CO_2_/cyclohexene oxide (CHO) ROCOP (TOF 10 000 h^−1^, 120 °C, 0.0025 mol %, 99 % PCHC).^[37]^ These catalyst‐tethered systems show a first order dependence on the overall catalyst concentration, whereas the analogous bicomponent system shows a fractional order in the metal complex.^[36]^ Another advantage is the improved selectivity for polymer, which is attributed to reduced backbiting from uncoordinated chains by electrostatic attractions “holding” any free anions “close” to the cationic catalyst. Neutral co‐catalysts were also tethered to metal complexes to deliver rate enhancements, for example, Lu and co‐workers applied a TBD‐tethered Co^III^‐salen complex that was six times more active than the bicomponent equivalent and maintained activity at low dilution (0.01 mol %).^[38]^

**Figure 6:**
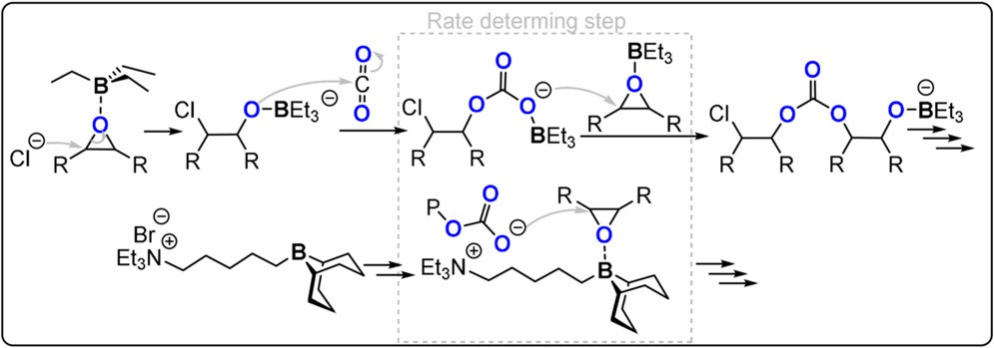
Proposed mechanism for bicomponent organocatalysed CO_2_/epoxide ROCOP.[^39^, ^40^]

**Figure 7:**
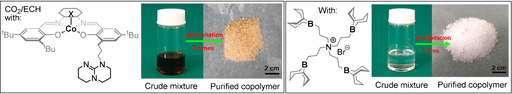
CO_2_/ECH copolymerisation by an ammonium‐borane catalyst produces a white polycarbonate.^[43]^ Copyright 2021 American Chemical Society.

Organocatalysts (although often featuring metals from Groups 1–13) are also active for CO_2_/epoxide ROCOP and are typically bicomponent systems comprising a Lewis acid/base pair. Some of these organocatalysts may be attractive in terms of ease of use on a small scale and their lack of colour. Feng and co‐workers discovered that Et_3_B (Lewis acid) and PPNCl (Lewis base) combinations showed good activity for both CO_2_/PO (TOF 49 h^−1^, 60 °C, 0.1 mol %, 10 bar, 83 % PPC) and CO_2_/CHO ROCOP (TOF 600 h^−1^, 80 °C, 0.025 mol %, 10 bar, 99 % PCHC).[^39^, ^41^, ^42^] DFT investigations support the rate‐determining step involving triethylborane epoxide and coordination of the propagating chain end (Figure 6); this mechanism closely resembles earlier reports for metal/co‐catalyst bimetallic pathways. Very recently, Wu and co‐workers reported a quaternary ammonium‐tethered 9‐borabicyclo(3.3.1)nonane, applied at 0.005 mol % loading, which shows high activity for CO_2_/CHO ROCOP (TOF 4900 h^−1^, 150 °C, 15 bar).^[40]^ A related ammonium salt quadruply tethered to borane moieties achieves CO_2_/epichlorhydrin (ECH) ROCOP to produce a white polymer (TOF 7 h^−1^, 25 °C, 0.1 mol %, 25 bar, 99 % polymer; Figure 7).^[43]^

Reports proposing bimetallic mechanisms for bicomponent systems motivated the preparation of di‐ and multi‐metallic catalysts, many of which operate without a co‐catalyst.^[18]^ In the best cases, these catalysts are as active as tethered catalyst/co‐catalyst systems and may be simpler to make and apply, since they maintain high activity at a much low CO_2_ pressure.^[43]^

**Figure 8:**
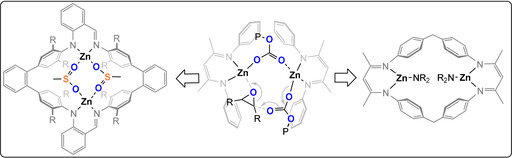
Bimetallic active site for CO_2_/CHO ROCOP proposed for zinc diketimide catalysts.[^21^, ^44^, ^45^]

Coates et al. pioneered Zn^II^‐diketimide catalysts (Figure 8) and through elegant kinetic investigations established the most active were loosely associated dimers; this excellent work has already been thoroughly reviewed.^[21]^ Lee et al. reported Zn^II^_2_‐bis(anilido‐aldimide) catalysts for CO_2_/CHO ROCOP (TOF 312 h^−1^, 80 °C, 0.02 mol %, 12 bar, 94 % PCHC) and noted that electron‐withdrawing substituents on the ligand enhanced the activity but reduced the selectivity (TOF 2860 h^−1^, 80 °C, 0.002 mol %, 14 bar, 79 % PCHC).[^44^, ^46^] Later, Rieger and co‐workers reported a highly active macrocyclic Zn^II^_2_‐bis(diketimide) catalyst (TOF 9130 h^−1^_,_ 100 °C, 0.025 mol %, 40 bar, >99 % PCHC).^[45]^ Curiously, it follows somewhat complex kinetics that are dependent on the carbon dioxide pressure, with a zero order at 5–25 bar changing to first order at 25–45 bar. Further studies showed that more rigid macrocycles reduced the activity,^[47]^ whereas electron‐withdrawing substituents increased it (TOF 155 000 h^−1^ 100 °C, 0.0125 mol %, 30 bar, 88 % PCHC).^[48]^

**Figure 9:**
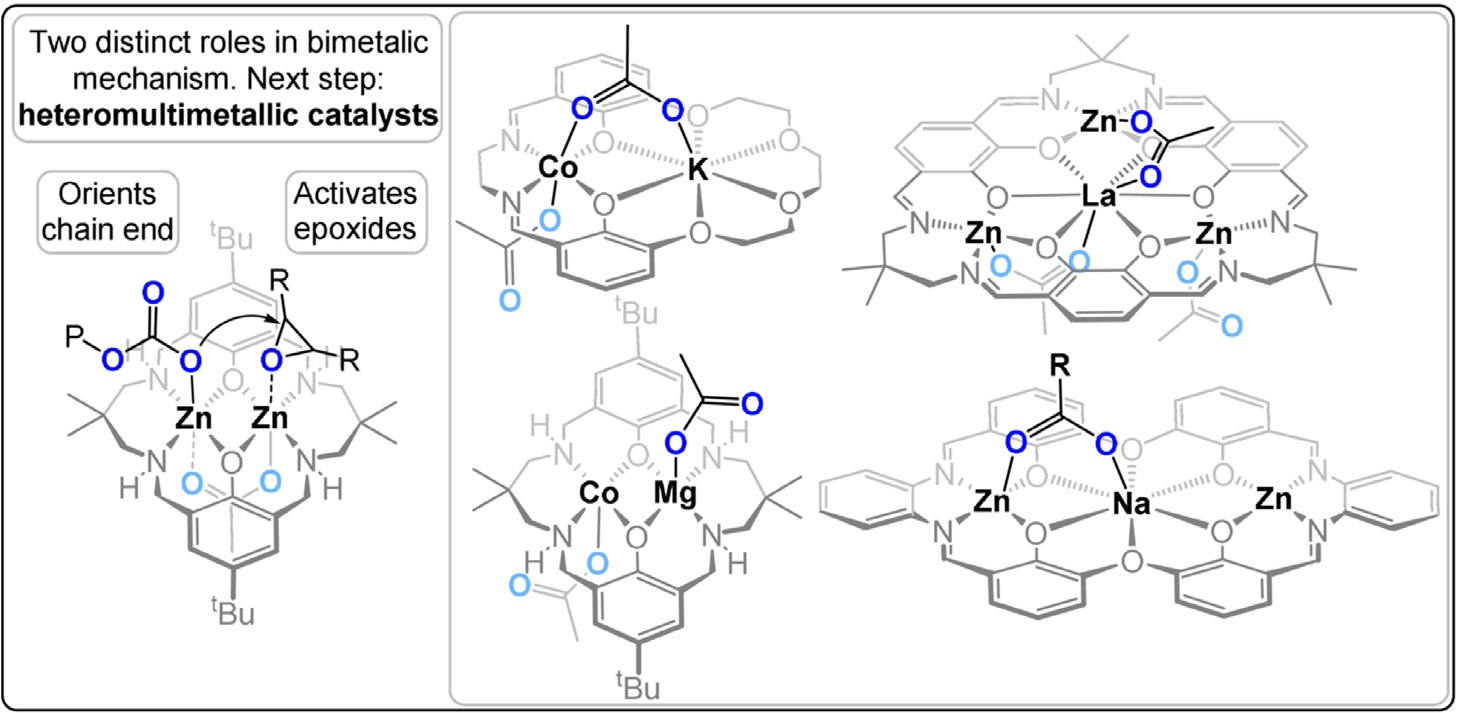
Heterodi‐ and multinuclear catalysts for CO_2_/epoxide ROCOP.[^53^, ^54^, ^55^, ^56^, ^57^]

Since 2008, our group has investigated catalysts featuring metal coordination to macrocyclic diphenolate tetraamine ligands.^[49]^ The first report described Zn^II^_2_ catalysts which showed moderate activity at 1 bar CO_2_ (TOF 18 h^−1^, 80 °C, 0.1 mol %) and were, at that time, a rare example of low‐pressure catalysts. Subsequently, Mg_2_ (TOF 35 h^−1^), Co^II^_2_ (TOF 161 h^−1^), and Fe^III^_2_ (TOF 6 h^−1^) catalysts have all showed activity at 1 bar CO_2_ (80 °C, 0.1 mol %).[^50^, ^51^, ^52^] Detailed investigations of the polymerisation kinetics, DFT calculations, in situ spectroscopy, and structure–activity studies supported a chain shuttling mechanism in which the polymer chain moves between the two metal centres with each monomer insertion.^[53]^ A key outcome from this mechanism was the potential for heterodinuclear catalysts, since each metal was attributed a distinct role in the catalytic cycle (Figure 9).

Heterodinuclear Zn^II^Mg^II^ catalysts showed greater activity than either homodinuclear analogue, that is, Zn^II^Zn^II^ or Mg^II^Mg^II^.[^58^, ^59^] This work provided the first evidence of catalytic synergy and supported the hypothesis that each metal has a distinct mechanistic function. Variants of this Zn^II^Mg^II^ catalyst, featuring organometallic, non‐initiating C_6_F_5_ co‐ligands, and applied with alcohol as a chain‐transfer agent, resulted in both high activity and selectivity for telechelic polycarbonates.^[60]^ The organometallic Zn^II^Mg^II^ catalysts react with the diols to form the desired alkoxide initiators in situ and deliver accurate control over the molar mass and end‐group chemistry. These catalysts were used to prepare polycarbonate‐*b*‐polyester‐*b*‐polycarbonate ABA triblock copolymers from mixtures of CHO, CO_2_, and bio‐based *ϵ*‐decalactone. The catalysts deliver precise control of the bock ratios and carbon dioxide contents (6–23 wt %). By controlling the carbonate linkage content, the material properties of the polymers were tuned from adhesives to elastomers to ductile plastics, thus addressing in particular the brittleness of the parent PCHC segments. In 2018, Mashima and co‐workers reported a series of high activity multimetallic catalysts featuring Zn^II^_3_Ln(III) for CO_2_/CHO ROCOP (TOF 300 h^−1^, 100 °C, 0.05 mol %, 3 bar CO_2_). In 2020 the same ligand was used to produce more active Co^II^_3_Ln(III) catalysts (TOF 1625 h^−1^, 0.004 mol %, 20 bar).[^56^, ^61^]

**Figure 10:**
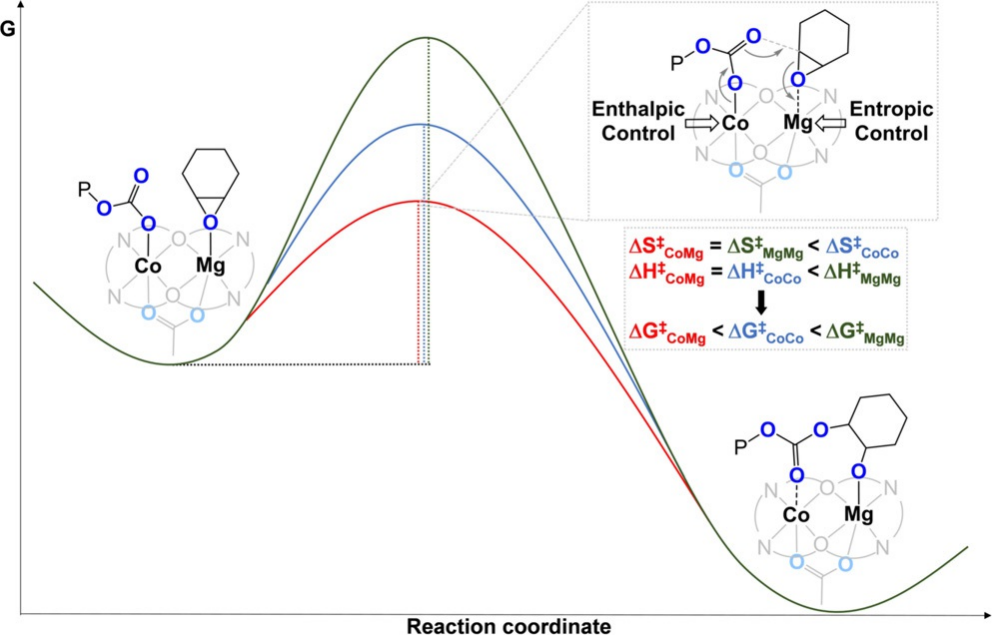
Co^II^Mg^II^ synergic heterodinuclear catalyst for CO_2_/CHO ROCOP compared with the Mg^II^Mg^II^ and Co^II^Co^II^ variants sheds light on the molecular basis for synergy.^[54]^

In 2020, our group investigated the phenomena which underpin the catalytic synergy in CHO/CO_2_ ROCOP by using a heterodinuclear Mg^II^Co^II^ catalyst (Figure 10).^[54]^ This catalyst showed an excellent activity at 1 bar (TOF 1205 h^−1^, 120 °C, 0.05 mol %, 99 % PCHC) and 20 bar CO_2_ pressure (TOF 12 460 h^−1^, 140 °C, 0.05 mol %, 99 % PCHC). It showed a rate four times higher than Mg^II^Mg^II^ and double that of Co^II^Co^II^ catalysts. Detailed kinetic analyses showed that |Δ*S* ^≠^| is reduced for Mg^II^‐containing catalysts, whilst Δ*H* ^≠^ is smaller for Co^II^‐containing variants. Hence, the success of the heterodinuclear catalyst is attributed to Mg^II^ coordinating the epoxide with a reduced transition‐state entropy, while the Co^II^‐carbonate attacks it with a reduced transition‐state enthalpy. Synergy arises because each metal has a distinct role in the catalytic rate‐determining step and the kinetics provide experimental evidence for this proposition. Epoxide ring opening transfers the propagating alkoxide to the Mg^II^ site and carbon dioxide insertion results in the chain “shuttling” back to the Co^II^ centre ready for the next cycle of monomer insertions.

In 2020, we also reported a heterodinuclear Co^III^K catalyst, coordinated by an asymmetric diphenolate, diamine macrocycle featuring a tetra‐ether moiety, which showed excellent activity in PO/CO_2_ ROCOP (TOF 800 h^−1^, 70 °C, 0.025 mol %, 30 bar CO_2_, 93 % PPC).^[62]^ Notably this catalyst tolerates up to 250 equivalents of chain transfer agent, and is thus useful for the production of polycarbonate polyols. Another heterotrimetallic catalyst featuring Zn_2_Na also shows good activity for CO_2_/CHO ROCOP at 1 bar CO_2_ and enables adjustable ether contents (TOF 75–956 h^−1^, 80–120 °C, 0.025 mol %, 5–33 % PCHO links in PCHC); this catalyst even retained good activity at 0.5 bar CO_2_ and can switch between CHO ROP and CO_2_/CHO ROCOP when changing the reaction atmosphere from CO_2_ to N_2_ and vice versa.^[57]^

**Figure 11:**
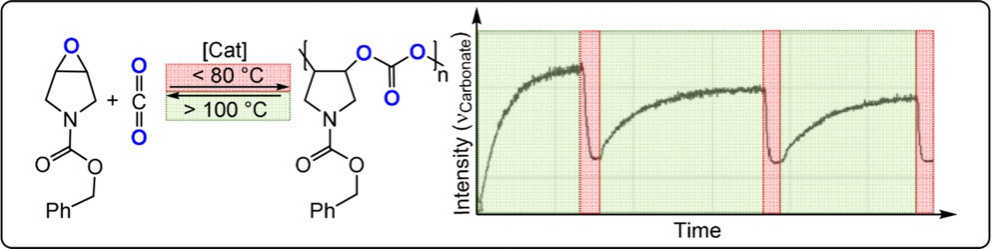
Thermally controlled reversible CO_2_/epoxide polymerisation and depolymerisation. Copyright 2017 Wiley.^[63]^

The polycarbonates prepared by CO_2_/epoxide ROCOP are usually the kinetic reaction products, which provides an opportunity to chemically recycle them to either cyclic carbonates or the parent monomers.^[3]^ Lu and co‐workers reported a di‐Cr^III^ catalyst for N‐heterocyclic epoxide/CO_2_ ROCOP which showed >99 % polymer selectivity at 60 °C (Figure 11).^[63]^ Nonetheless, at 100 °C, a near quantitative depolymerisation occurred, re‐forming the epoxide and CO_2_. Depolymerisation of the purified polymer back into monomers also occurred in the bulk phase at higher temperature (260–300 °C) without any catalyst. This result illustrates future potential in circular polymerisation/depolymerisation process, although it should be emphasised that such a low‐temperature depolymerisation could be problematic for polymer processing and may need optimization. Fully bio‐derived poly(limonene carbonate), prepared by CO_2_/limonene oxide (citrus fruit peel) ROCOP, was also depolymerized to limonene oxide and CO_2_ using either a Zn^II^_2_ complex or organic bases.[^64^, ^65^] This depolymerisation is both monomer‐ and catalyst‐dependent, since reports of PCHC depolymerisation indicate selective c5c formation,[^57^, ^66^, ^67^, ^68^] although, *trans*‐c5c can undergo ring‐opening polymerization to form polycarbonates.[^69^, ^70^] Accordingly, Coates and co‐workers reported a neat proof of chemical recycling using isotactic PCHC, synthesized using an enantioselective Zn^II^‐bis(diketimide) catalyst. Vacuum thermolysis at 250 °C selectively depolymerized it into *trans*‐c5c in 95 % yield; the cyclic carbonate was subsequently efficiently repolymerized.^[68]^

## 3 Recent Trends in Anhydride/Epoxide ROCOP

**Figure 12:**
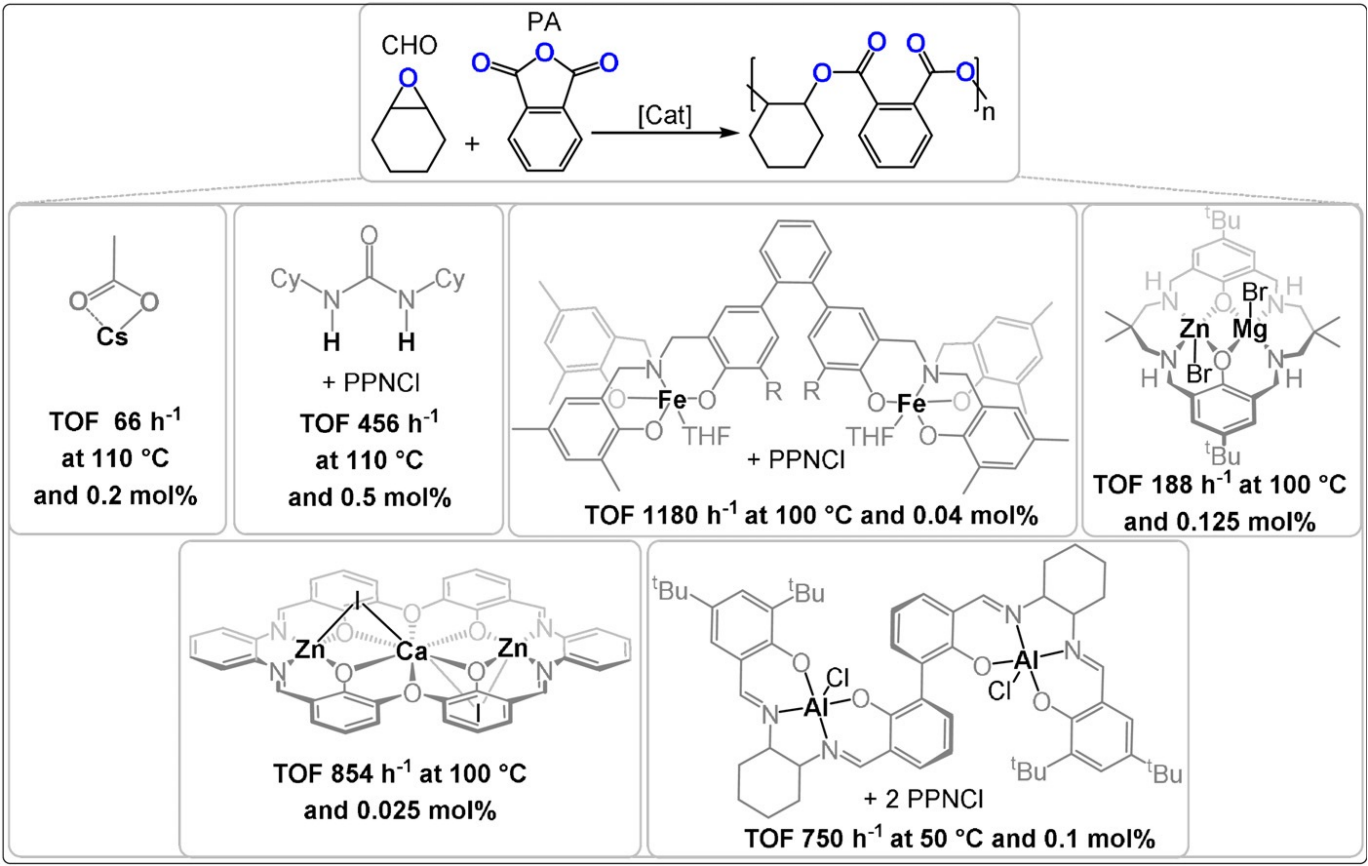
Selection of high‐performance CHO/PA ROCOP catalysts.[^57^, ^58^, ^72^, ^73^, ^74^, ^75^]

Another classic ring‐opening copolymerisation is that of cyclic anhydrides with epoxides to yield polyesters.^[13]^ It enables access to many different polyester backbone and side‐chain reactions. In contrast to heterocycle ROP, the ring strain of epoxides/anhydrides is less impacted by substituents and, thus, the polymerisation remains thermodynamically feasible using substituted/functionalized monomers.^[71]^ It is also an excellent means to increase backbone “rigidity” through the incorporation of aromatic or strained heterocyclic units. Many epoxides and anhydrides are already large‐scale chemical products and this may help accelerate the implementation of this polymerisation method. There have already been some comprehensive reviews on epoxide/anhydride ROCOP; here only recent developments in catalysis will be described, with a focus on findings most relevant to other monomer combinations.[^8^, ^9^] Here, most catalysts are benchmarked by performances using phthalic anhydride(PA)/CHO, but so far this field lacks common standards and the multitude of other monomers and reaction conditions complicate comparisons of the catalysts (Figure 12).

Most catalysts active for CO_2_/epoxide are also active for anhydride/epoxide ROCOP, but the reverse isn't necessarily true. For example, Lewis base catalysts only form c5c with CO_2_/epoxides, but catalyse anhydride/epoxide ROCOP at appropriate temperatures.[^76^, ^77^] This reactivity difference stems from the side reactions: alkoxide‐terminated polymer chain ends back‐bite into adjunct carbonate links to form c5c, but such a pathway has a higher barrier in the case of anhydride/epoxide ROCOP. Nevertheless, in the latter polymerisation, alkoxide chain ends can undergo transesterification, thereby broadening the molar mass distributions.

**Figure 13:**
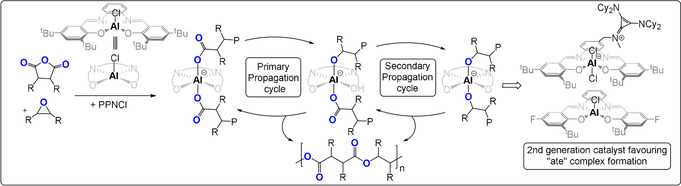
Coates and co‐workers applied catalyst design to minimize transesterification side reactions.[^78^, ^79^, ^80^]

Tolman, Coates, and co‐workers reported an excellent ROCOP mechanistic investigation using Al^III^‐salen/PPNCl bicomponent catalysts (Figure 13).^[78]^ A bis(carboxylate) aluminate resting state was proposed in the initial stages of the catalysis, even when a large excess of epoxide was present. The rate‐determining step was proposed as epoxide insertion into the aluminium‐carboxylate intermediate to produce a mono(alkoxide)‐mono(carboxylate) aluminate intermediate. Rapid insertion of an anhydride monomer into this intermediate regenerated the bis(carboxylate) aluminate resting state. The Al^III^‐salen/PPNCl catalyst forms an ion pair and should be treated as such in any kinetic analyses. As the polymerisation progressed and the anhydride concentration became depleted, the bis(alkoxide) aluminate intermediate accumulated and undesired side reactions, such as transesterification, became feasible. Inspired by the mechanism, Coates and co‐workers reported highly active aminocyclopropenium chloride tethered Al^III^‐salen catalysts which maintained high activities at low catalyst loading (PO/norbornene anhydride, TOF 80 h^−1^, 60 °C, 0.005 mol %, vs. TOF 10 h^−1^ for a bicomponent catalyst).^[79]^ These properly designed tethered catalysts showed much less transesterification, epimerisation, and chain‐end coupling reactions than bicomponent analogues. The performances were rationalised by control over the metalate equilibria avoiding formation of free alkoxide chains. The tethered catalyst was also tolerant of large quantities of chain transfer agent, thereby allowing control of the molar mass.^[81]^ In bicomponent systems, chain transfer agents reduce the propagating chain nucleophilicity, through hydrogen bonding, and suppress metalate formation through competitive coordination to the metal centre. The tethered system intrinsically favours metalate formation and hence counterbalances deleterious influences of chain transfer agents and allows access to branched and star polymers.

Coates and co‐workers also developed Al^III^‐salen catalysts with electron‐withdrawing *para*‐fluoro substituents, which significantly reduced transesterification.^[80]^ It was proposed that the substituents stabilize the aluminate and prevent dissociation of the alkoxide chain end from the catalyst, a hypothesis reminiscent of those rationalizing the enhanced performances of tethered catalysts systems in CO_2_/epoxide ROCOP.

## 4 ROCOP of CO_2_ with Oxetanes

**Figure 14:**
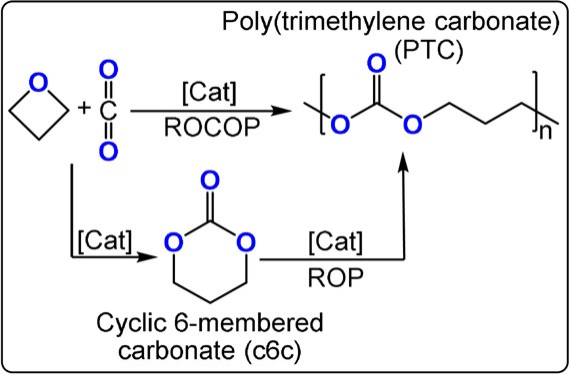
Copolymerizations of CO_2_ and oxetane proceed either by direct CO_2_/oxetane ROCOP or via intermediate c6c and its ROP into PTC.

The four‐membered cyclic ether oxetane shows only a slightly lower ring strain (Δ*H*=81 kJ mol^−1^) than its three membered cousin, ethylene oxide (Δ*H*=104 kJ mol^−1^): its copolymerisation with carbon dioxide should, therefore, be feasible.^[82]^ Following initial reports of low‐yielding ternary catalysts, Baba and co‐workers reported bicomponent Lewis acid (Bu_2/3_SnI_2/1_)/(phosphine, nitrogen Lewis base) catalysts (TOF ca. 7 h^−1^, 100 °C, 2 mol %, 49 bar, *M_n_* ≈1200 g mol^−1^, 88–99 % carbonate content).[^83^, ^84^, ^85^] The mechanism was ambiguous with respect to the formation of trimethylene carbonate (c6c) as either an intermediate or by‐product, because c6c ROP is feasible and forms the same poly(trimethylene carbonate) (PTC; Figure 14).^[86]^ The authors observed an initial increase in the c6c concentration that correlated with the decreasing concentration of oxetane, but was followed by a decrease in the c6c concentration over longer reaction times and the formation of PTC.

**Figure 15:**
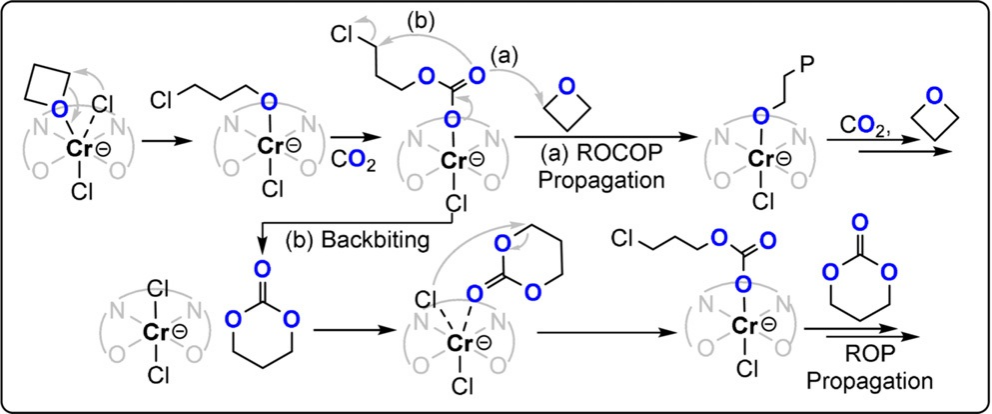
CO_2_/oxetane ROCOP catalysed by a Cr^III^‐salen/R_4_NCl catalyst, with different direct and indirect pathways illustrated.^[87]^

**Figure 16:**
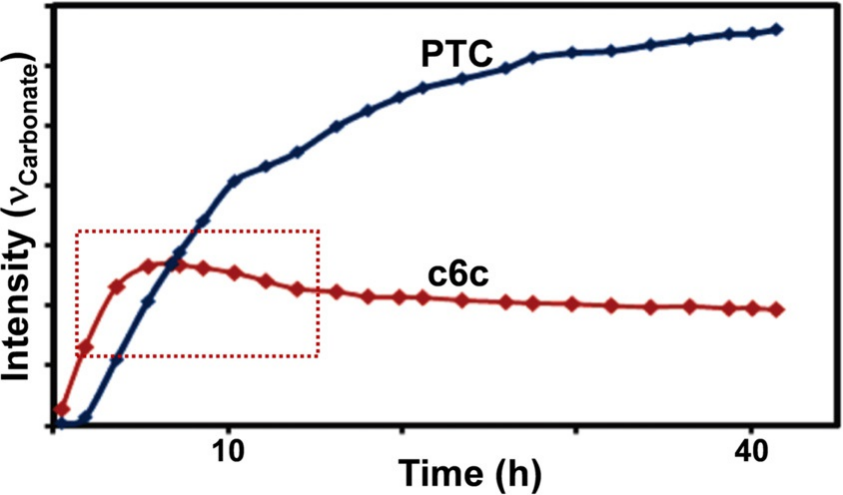
In situ IR spectroscopy applied to CO_2_/3‐methoxymethyl‐3‐methyloxetane ROCOP using Cr^III^‐salen/R_4_NN_3_. Data show a maximum c6c concentration (highlighted in red) which supports a parallel c6c ROP mechanism. Copyright 2011 American Chemical Society.^[92]^

The most active catalyst system is Cr^III^‐salen/R_4_NCl (TOF 41 h^−1^, 110 °C, 0.08 mol %, 35 bar, *M_n_* ≈10.1 kg mol^−1^).^[88]^ It's activity was lower for copolymerisations with oxetane than epoxides, but its selectivity for polymer versus cyclic carbonate was much higher. It was proposed that c6c ROP and CO_2_/oxetane ROCOP operate simultaneously, with the active catalyst being a chromate species (Figure 15). The overall rate law was first order in the concentrations of the catalyst ion pair (i.e. chromate) and oxetane; the experimental Δ*G* ^≠^_ROP_=101.9 kJ mol^−1^ and Δ*G* ^≠^_ROCOP_=107.6 kJ mol^−1^.[^89^, ^90^, ^91^] Copolymerisation of 3,3′‐substituted oxetanes was also feasible, but slower than for oxetane and also occurred through c6c ROP (Figure 16).^[92]^ Substituted oxetanes generally formed more c6c in the final product mixtures, likely due to equilibrium constraints on their ROP. The catalyst also readily depolymerised disubstituted PTC when applied without a CO_2_ atmosphere.

**Figure 17:**
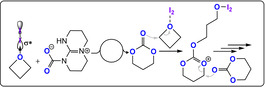
CO_2_/oxetane ROCOP using a bicomponent I_2_/TBD catalyst.^[94]^

Ammonium salts R_4_NX (X=Cl, Br, I, N_3_, OAc) with a bis‐hydrogen bond donor, such as I_2_ or BEt_3_, were also active catalysts (Figure 17).[^93^, ^94^, ^95^, ^96^] It was proposed that oxetane activation occurred by borane coordination, hydrogen bonding, or halogen bonding. Some organocatalysts were effective when using unpurified monomers, although the resulting molar masses of the polycarbonates were very low (<2 kg mol^−1^), likely due to contamination by a protic compound. Although readily available, all these organocatalysts required high loadings of 0.5–3 mol %, show only modest activities (TOF ≤5 h^−1^, ≥90 °C), and produced variable c6c/PTC product ratios depending on the reaction conditions.

Improving catalyst performances as well as tackling monomer purity issues to drive up molar mass values (both of which are lagging behind what has been achieved with CO_2_/epoxide) will be essential to yield useful PTC which, when prepared by other means, shows promise in biomedical applications.[^97^, ^98^] PTMC (*T*_g_ ca.−20 °C) has been used to form matrices for cell growth, with a particular focus on the regeneration of bone, cartilage, nerve, and/or blood vessels.[^99^, ^100^, ^101^, ^102^] PTC undergoes rapid hydrolysis under biologically relevant conditions, but unlike aliphatic polyesters does not generate acidic decomposition products and thus can reduce inflammation side effects.^[103]^ Such properties are important for any biomedical implants targeted to fully degrade after healing.^[104]^ As a component in block polymers, it also allows the controlled release of anti‐cancer drugs, proteins, and gene‐therapeutics.[^105^, ^106^, ^107^]

## 5 Other ROCOPs Involving Oxetanes and Tetrahydrofuran

Oxetane ROCOP with cyclic anhydrides is also feasible but under‐investigated. Endo and co‐workers described Ti^IV^ bis(phenolate) catalysts that were active in the presence of various anhydrides, although in some cases around 40 mol % ether linkages were observed (TOF <3 h^−1^, 1.7 mol %, 120 °C, *M_n_* =2.8–4.9 kg mol^−1^).^[108]^ Adding a phosphonium co‐catalyst resulted in completely alternating polymers from 3,3′‐disubstituted oxetanes and succinic/diphenic anhydride (TOF <1 h^−1^, 130 °C, 5 mol %, *M_n_* =2.9–11.1 kg mol^−1^).^[109]^ Alternating copolymers from anhydride/THF ROCOP were achieved with a bistriflimidic acid catalyst (TOF 3–10 h^−1^, 120 °C, 1 mol %, *M_n_* =2.0–3.5 kg mol^−1^).^[110]^ The organoaluminium catalyst Al(*i*Bu)_3_ also terpolymerises anhydrides, epoxides, oxetane, or THF into (ABC)_*n*_ polymers.^[111]^ Although the properties of the resulting polyesters remain under‐investigated, increasing the chain length between the ester linkages is expected to accelerate biodegradation and may hold future promise for tailoring degradation rates.^[112]^

## 6 ROCOP of Dihydrocoumarin with Epoxides

**Figure 18:**
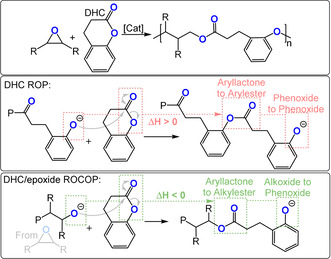
Dihydrocoumarin (DHC)/epoxide ROCOP by different pathways.

The copolymerization of aliphatic lactones and epoxides may lead to the formation of copolymers (block/random/gradient) but rarely yields alternating copolymers selectively.[^113^, ^114^, ^115^] In contrast, the semi‐aromatic lactone DHC does not undergo ring‐opening polymerisation because of its low ring strain and chain‐end reactivity (Figure 18). One way to overcome this barrier would be if DHC were ring‐opened by an alkoxide chain end, such as that derived from epoxide ring opening. In such a reaction, an aryl‐alkyl‐lactone becomes an alkyl‐alkyl‐ester and the chain end is a mesomerically stabilized phenoxide. Hence, DHC/epoxide ROCOP is an exergonic process. DHC is an attractive monomer as it is comparatively inexpensive (0.03 $ g^−1^), can be sourced from renewable coumarin, and is “generally considered safe” by the FDA.^[116]^

Endo and co‐workers pioneered DHC/glycidyl ether ROCOP, using imidazole catalysts, to produce polyesters with 99 % selectivity, moderate activity, and low molar mass (TOF 30–50 h^−1^, 120 °C, 2 mol %, *M_n_* <3 kg mol^−1^, 94–99 %).[^117^, ^118^, ^119^, ^120^] Using a bifunctional DHC, in which two lactones were connected through a benzene core, copolymerisation with epoxides resulted in polyesters featuring pendant lactones. These lactones were post‐functionalized through alcoholysis/aminolysis reactions, or provided sites for cross‐linking reactions, which resulted in materials showing higher *T*_g_ values (60 °C vs. 71–112 °C, *M_n_* =1.5–3 kg mol^−1^). Furthermore, this DHC/epoxide‐based curing system undergoes minimal volume shrinkage—a valuable attribute for bonding materials in electronic devices.[^121^, ^122^]

Using a Cr^III^‐salen/PPNCl catalyst system improved the reaction rates (TOF 7–42 h^−1^, 80 °C, 0.2 mol %, *M_n_* =7–20 kg mol^−1^).^[123]^ Strangely, many successful CO_2_ or anhydride/epoxide ROCOP catalysts, for example, bis(diketimide)ZnOAc or Co^III^‐salen/PPNCl, were significantly less active in DHC/epoxide ROCOP. Nonetheless, the Cr^III^‐salen catalyst system showed broad epoxide scope and yielded polyesters with glass transition temperatures from −8 to 57 °C. Most polyesters were amorphous, but DHC/cyclopentene oxide (CPO) or DHC/CHO ROCOP yielded semi‐crystalline but atactic polyesters showing moderate melting temperatures (DHC/CPO: *T_m_* =139 °C, DHC/CHO: *T_m_* =173 °C). Lu and co‐workers reported the highest activity catalyst—a binuclear [Cr^III^‐salen]_2_/PPNCl system (TOF 11–115 h^−1^, 30–100 °C, 0.1 mol %, *M_n_* =5–19 kg mol^−1^).^[124]^ Under equivalent conditions, it was significantly more active in CO_2_ or PA/CHO ROCOP than DHC/CHO ROCOP. These differences in reactivity were exploited to deliver block polyesters (poly((PA‐*alt*‐CHO)‐*b*‐(DHC‐*alt*‐CHO)) from mixtures of PA, DHC, and CHO. Organocatalysts comprised of phosphazene bases and alcohols were also active for DHC/epoxide ROCOP (TOF <2 h^−1^, 50–80 °C, 2–0.2 mol %, *M_n_* =2.5–12 kg mol^−1^), although much slower than Cr‐based catalysts and, furthermore, they were less selective and resulted in greater transesterification.[^125^, ^126^] It was proposed that proton exchange reactions between the chain end and the phosphazene base allowed alcohols to act as chain transfer agents. Under such conditions, up to 10 equivalents of alcohol (vs. phosphazane base) were used to make branched or brush polymer architectures.

## 7 ROCOP of γ‐Chalcolactone with Epoxides and Thiiranes

**Figure 19:**
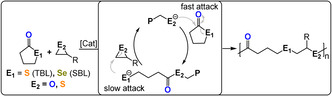
ROCOP between thio‐/selenolactones and epoxides or thiiranes.

γ‐Butyrolactone and its chalcogen derivatives, γ‐thiobutyrolactone (TBL) and γ‐selenobutyrolactone (SBL), have very low ring strain and so ROP is thermodynamically unfeasible under most conditions.^[127]^ This means that these monomers are suitable candidates for ROCOP and, in fact, TBL/epoxide ROCOP was feasible using R_4_N/KCl salt catalysts (TOF 3–31 h^−1^, 90 °C, 3 mol %, *M_n_* =7.7–9.5 kg mol^−1^) or phosphazene bases with alcohols (TOF <5 h^−1^, 25–60 °C, 4 mol %, *M_n_* =2–6 kg mol^−1^) and yielded poly(ester‐*alt*‐thioether) (Figure 19).[^128^, ^129^] The ROCOP kinetics indicated rapid TBL insertion but slow epoxide ring‐opening, thereby resulting in a thiolate resting state.

The use of the selenium analogue (SBL) resulted in fast ROCOP for many epoxides when using phosphazene bases with alcohols (TOF 68–1455 h^−1^, 25 °C, 1 mol %, *M_n_* =2.1–21.1 kg mol^−1^) or R_4_N‐halide salts (TOF 2 h^−1^, 80 °C, 2 mol %, *M_n_* =4.6–6.8 kg mol^−1^).[^130^, ^131^] The poly(ester‐*alt*‐selenoether)s were amorphous with low glass transition temperatures (*T_g_* =−59 to −5 °C, *T_d_* =227–275 °C) and high refractive indices (RI >1.6). This method was also used to make block polymers by sequential epoxide additions. Thiirane/SBL ROCOP to form poly(thioester‐*alt*‐selenoether) was catalysed by phosphazene bases with thiols (TOF ca. 1200 h^−1^, −20 °C, 1 mol %, *M_n_* =2.5–12.6 kg mol^−1^, *T_g_* =−45 °C for propylene sulfide (PS); TOF 200 h^−1^, 25 °C, *M_n_* =3.5–12.8 kg mol^−1^, *T_g_* =−4.2 °C for cyclohexene sulfide (CHS)).^[132]^ Low polymerisation temperatures were required to avoid homopolymerisation, since the barrier to thiirane ROP is quite low (e.g 14.8 kJ mol^−1^ for PS).

Polymers containing sulfur or selenium links can be oxidative responsive materials. For example, block copolymers containing a chalcogen‐containing hydrophobic block with a hydrophilic block self‐assembled into micelles when dispersed in water. Upon oxidation with, for example, H_2_O_2_, the chalcogen centres become hydrophilic, thereby causing the micelles to disassemble, as demonstrated with a block polymer prepared by SBL/epoxide ROCOP.[^131^, ^133^, ^134^] This property was explored for drug delivery in a recent report of a polymer containing selenoether and PEG blocks. These micelles were loaded with doxorubicin and a photo‐oxidant, with the anti‐cancer drug released upon irradiation with IR light.^[135]^

## 8 ROCOP of Bicyclic Butyrolactones with Epoxides

**Figure 20:**
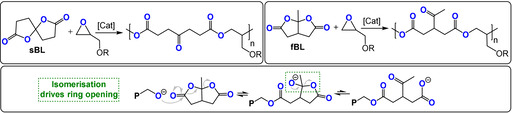
Bicyclic butyrolactones (left: spiro‐butyrolactone (sBL); right: fused bis‐butyrolactone (fBL))/epoxide ROCOP by different pathways.

Endo and co‐workers also developed a useful method to copolymerize bicyclic butyrolactones with epoxides.[^136^, ^137^, ^138^, ^139^, ^140^, ^141^, ^142^, ^143^, ^144^] Accordingly, either fused bicyclic butyrolactones (fBL) or spirocyclic butyrolactones (sBL) were copolymerized with glycidyl ethers (Figure 20). The ring opening of the bicyclic monomer is driven by a thermodynamically favoured isomerisation to a ketone. In terms of catalysts, phosphines were the most active and selective (TOF 1–10 h^−1^, 120 °C, 0.8 mol %, *M_n_* =5.2–6.7 kg mol^−1^). The sBL/phenyl glycidyl ether (PGE) copolymer exhibits a *T_g_* of 60 °C (*T_d_* =287 °C), which is significantly higher than that of the fBL/PGE copolymer with a *T_g_* of −15 °C (*T_d_* =356 °C)—these differences were attributed to internal versus pendant ketone groups modulating the chain flexibility.

The copolymerisation of either sBL or fBL resulted in volume expansion, a consequence of the double ring opening of the fused/spiro cycle, which may be useful for polymer resins.^[140]^ Whereas epoxide ROP results in volume shrinkage, isopropyl‐fBL/PGE ROCOP had the same volume before and after polymerisation. In resin applications, shrinkage is undesirable, especially for adhesive or filler uses, and may lead to sub‐optimal interfaces and performances. Endo and co‐workers also reported isoprene‐substituted bBL which, after ROCOP with PGE, was functionalized through thiol‐ene reactions or radically cross‐linked to form networks.^[142]^ The latter networks showed increased rigidity and the expected improvement in thermal stability (which increased by 50 °C to give *T_d_* =326 °C).

## 9 ROCOP of COS with Epoxides and Oxetane

Carbonyl sulfide (COS), the monosulfur analogue of CO_2_, is both a naturally occurring gas (e.g. released by marine plants or volcanic eruptions) and an anthropogenic environmental pollutant emitted by burning fossil fuels.[^145^, ^146^] It is a major source of acid rain as it can be oxidized to SO_2_ in the troposphere and, furthermore, damages the ozonosphere. Therefore, its use as a monomer could redress the environmental impact and valorise an industrial waste.[^147^, ^148^]

**Figure 21:**
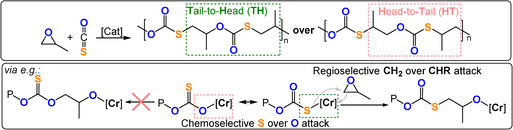
COS/epoxide ROCOP illustrating different chemo‐ and regioselectivities.^[149]^.

In 2013 Zhang and co‐workers successfully achieved both chemo‐ and regioselective COS/PO ROCOP using a Cr^III^‐salen/PPNCl catalyst system to produce monothio‐PPC (TOF 288–332 h^−1^, 25 °C, 0.1 mol %, *M_n_* =21.9–25.3 kg mol^−1^).^[149]^ The selectivity for monothiocarbonate (‐(S‐)C(=O)‐O‐) linkages was attributed to the preferential coordination of sulfur (rather than oxygen) to Cr^III^. The increased nucleophilicity of the Cr^III^‐thiolate intermediate (compared with Cr^III^‐alkoxides) was attributed to the higher activities compared to those for CO_2_/PO ROCOP. The thiocarbonate linkage is asymmetric, so copolymerisation with monosubstituted epoxides may form four regioisomeric linkages: HT, TH, TT, and HH. The notation describes whether the CH_2_ (T) or CHR (H) group sits adjacent to the respective sides of the monothiocarbonate links (Figure 21); the Cr^III^‐salen catalyst system showed remarkably high selectivity for TH linkages (>98 %). The polymerisation conditions needed to be quite finely balanced, since a moderate increase in the temperature (60 °C) or adventitious moisture formed cyclic carbonate, dithiocarbonate (‐SC(=O)S‐), and carbonate (‐OC(=O)O‐) linkages by O/S scrambling.^[150]^ The Cr^III^‐salen/PPNCl catalysts also showed good activity in COS ROCOP with other epoxides, for example, COS/CHO (TOF 325 h^−1^, 40 °C, 0.1 mol %, *M_n_* =12.3 kg mol^−1^), COS/PGE (TOF 7300 h^−1^, 25 °C, 0.1 mol %, *M_n_* =22.6 kg mol^−1^), and COS/styrene oxide (TOF 83 h^−1^, 20 °C, 0.1 mol %, *M_n_* =77.2 kg mol^−1^).[^151^, ^152^, ^153^]

The glass transition temperatures of the poly(monothiocarbonates) are similar to those of the polycarbonate analogues and can be easily controlled over a wide temperature range by changing the epoxide (*T_g_* =3–115 °C). Many polymers are optically transparent, with high refractive indices, for example, RI=1.63 for COS/PO or 1.70 for COS/CHO.^[147]^ To avoid chromatic aberration in optical applications, the RI should remain constant as the refracted light wavelength changes, a property expressed by the Abbe number *V_d_* (i.e. higher *V_d_* values are better for uses as lenses, prisms, or waveguides).^[154]^ In COS/epoxide ROCOP, *V_d_* was controlled, and could be increased, by forming random polymers through terpolymerisation. For example, COS/PO/CHO ROCOP leads to tuneable *V_d_* values (32.1–43.1), high RI values (1.52–1.56), and *T_g_* =44–93 °C, depending on the CHO/PO feed ratios.^[152]^ CHO/CO_2_/COS ROCOP, using an [Al^III^ salen]_2_/PPNCl catalyst, gave polymers with the highest *V_d_* value of 48.6 for randomized equimolar carbonate/thiocarbonate links while maintaining high glass transition and decomposition temperatures (*T_g_* =111 °C and *T_d_* =260 °C).^[155]^

As was also observed for CO_2_/epoxide ROCOP, tethered catalysts showed the highest activity and selectivity as well as excellent performance at the lowest catalyst loadings. For example, a DBU‐tethered Cr^III^‐salen catalyst operates at high polymerisation temperatures, thereby resulting in very high activity without compromising the O/S scrambling or polymer selectivity (TOF 4670–271 000 h^−1^, 25–80 °C, 0.005–0.00005 mol %, 27.1–220.0 kg mol^−1^).^[156]^ In direct contrast to CO_2_/PO ROCOP, where Co^III^ catalysts were usually more active than Cr^III^ systems, the reverse was observed for COS/PO ROCOP. The Cr^III^‐salen catalyst was also active for other COS/monosubstituted epoxide ROCOP reactions, and always showed high activity (TOF >20 000 h^−1^). Two slower copolymerisations—COS/CHO (260 h^−1^) and COS/CPO ROCOP (1360 h^−1^)—were accelerated when 1 mol % PO was added (TOF 15 800 h^−1^). The heterogeneous Zn/Co^III^‐DMC catalysts were also active in COS/CHO ROCOP (190 g g^−1^ h^−1^, 110 °C, *M_n_* =6.5–25.0 kg mol^−1^), forming colourless polymers (c.f. the Cr^II^‐salen/PPNCl catalysts yielded pale yellow polymers even after repeated washing), although with 10 % O/S scrambled linkages.^[157]^

**Figure 22:**
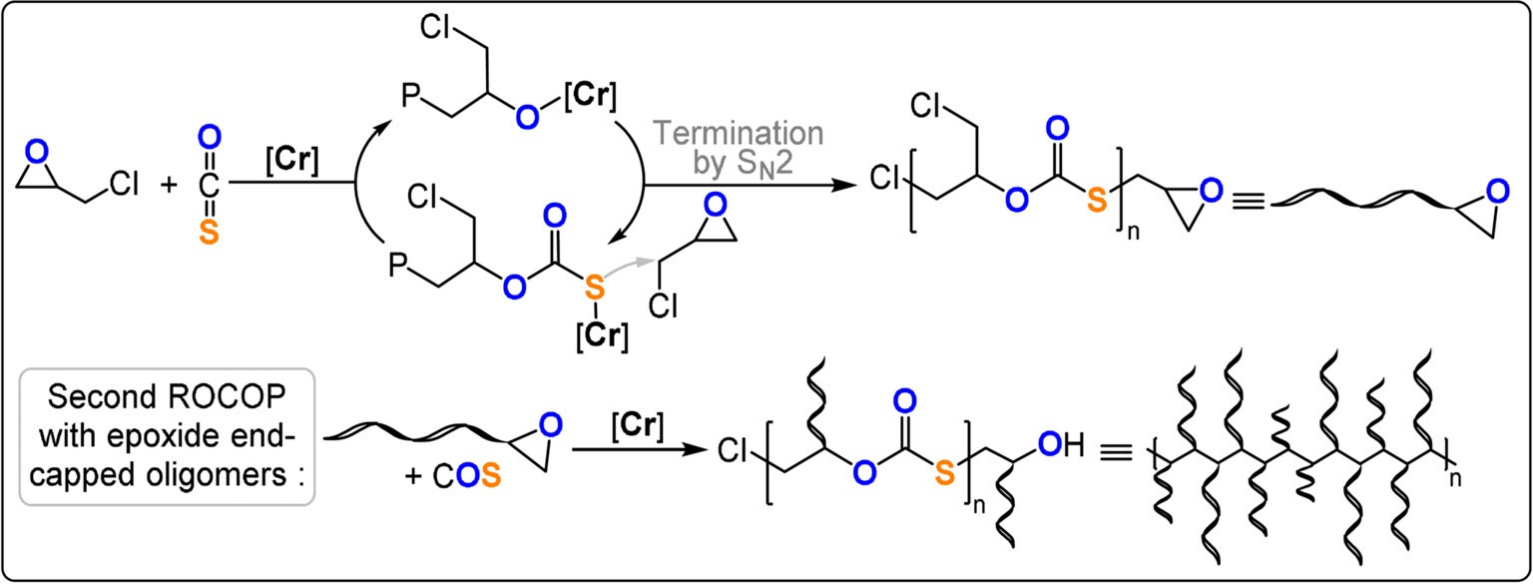
Semicrystalline graft polymers produced by two‐stage COS/ECH ROCOP.^[158]^

Recently semicrystalline poly(monothiocarbonates), derived from COS/epoxide ROCOP, were made by polymerizing enantiopure ECH with COS using a DBU‐tethered Cr^III^‐salen catalyst (TOF 19 h^−1^, −25 °C, 0.1 mol %, *M_n_* =3.1 kg mol^−1^, *T_g_* =16 °C, *T_m_* =97 °C).^[159]^ The polymerisation suffered from termination reactions, with the molar mass values being very low, and these occurred by nucleophilic substitution reactions between the growing polymer chain and the chloride substituent of ECH (Figure 22). Nonetheless, these epoxides underwent a second COS/epoxide ROCOP to yield semi‐crystalline graft polymers.^[158]^ In a two‐step process, the length of the substituent on the epoxide‐terminated “macromonomer” was initially determined by the reaction temperature (−20 to 0 °C). In the second step, the temperature was increased to 25 °C and the macromonomer underwent ROCOP to form graft polymers (*M_n_* =32.9–37.8 kg mol^−1^, with 1.5–1.8 kg mol^−1^ branches). Both ROCOP processes were highly controlled and about 90 % ECH was converted before formation of the graft polymer. Naturally, the graft polymer showed different thermal properties to the starting macromonomers (*T_g_* =11 °C, *T_m_* =113 °C, *T_d_* =262 °C). As graft polymers could be readily formed by ROCOP, either with multi‐functional chain transfer agents or through temperature switching, this might be a promising future direction for these polymers.^[23]^ Generally, graft polymers show different rheology and viscosity compared to their linear components because of different chain entanglement. Therefore, branched polymer solutions are often much less viscous than their linear counterparts, which may facilitate processing and applications, for example, in the administration of liquid drug formulations which are thus easier to inject by syringe.[^160^, ^161^] Branched polymers can host a guest molecule through non‐covalent encapsulation within the cavities between the chains—a relevant feature for the delivery and controlled release of pharmaceuticals.^[162]^

**Figure 23:**
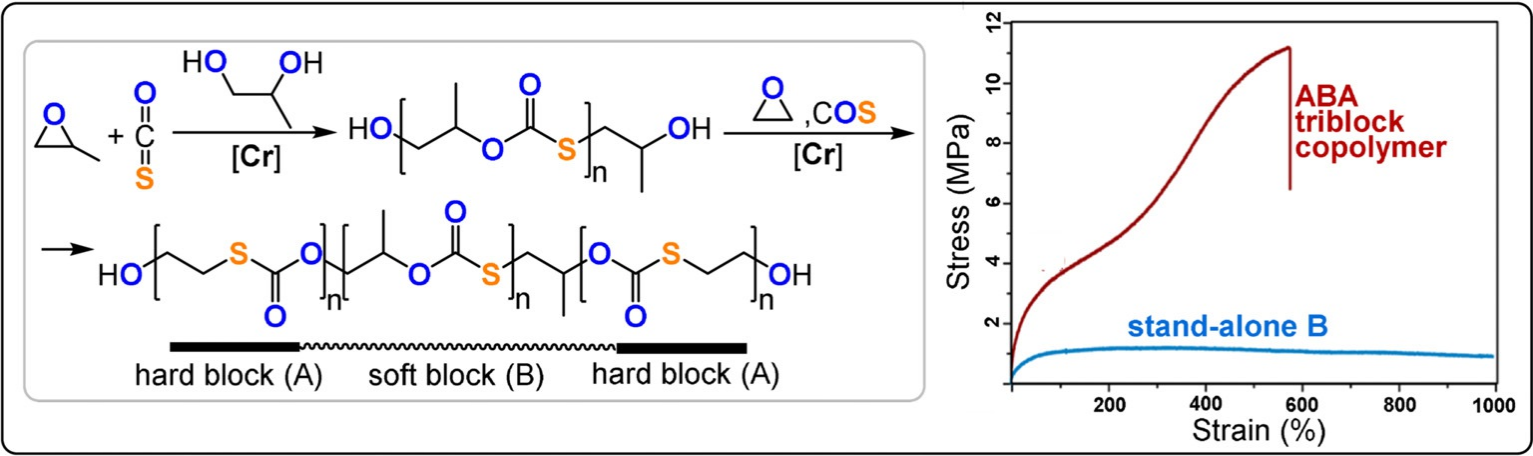
Synthesis of ABA thermoplastic elastomers and comparison of their mechanical properties with a B homopolymer. Copyright 2017 American Chemical Society.^[163]^

Isotactic COS/PO and COS/PGE copolymers, prepared from enantiopure epoxides, were amorphous, but the COS/EO polymer was semicrystalline (*T_m_* =128 °C, *T_c_* =66 °C; note that the all‐oxygen variant poly(ethylene carbonate) derived from CO_2_/EO ROCOP is amorphous (*T_g_* =0–10 °C).[^149^, ^152^, ^158^] The catalyst system was a DBU‐tethered Cr^III^‐salen complex and it showed very high activity (TOF 84 900 h^−1^) to produce a high molecular weight polymer (*M_n_* =193 kg mol^−1^). An ABA triblock polymer (COS/EO‐*b*‐COS/PO‐*b*‐COS/EO), which features a semicrystalline‐soft‐semicrystalline combination (30 % COS/EO units, 70 % COS/PO units), with a moderate molecular weight (*M_n_* =13 kg mol^−1^) showed a stress at breaking of 11.2±0.1 MPa and an elongation at breakage of 575±52 %.^[163]^ The thermoplastic elastomer showed an elastic recovery of about 90 % after five 300 % strain cycles; although the performance cannot match existing thermoplastics, for example, styrene/butadiene SBS (28 MPa stress and 800 % elongation at break) or polyurethanes PU (25–75 MPa stress and 500 % elongation at breaking point), it serves as proof of potential (Figure 23).^[164]^

Lu and co‐workers employed an asymmetric catalyst to make semi‐crystalline poly(monothiocarbonates) from achiral *meso*‐epoxides and COS.^[165]^ In the case of COS/CHO ROCOP, [Cr^III^ salen]_2_ yielded atactic polymer, but the Co^III^ analogue produced highly isotactic polymer (*P_m_* =90 %). The extent of the isotacticity of course controlled the maximum melting temperature, with values up to 201 °C observed at 99 % isotacticity (*M_n_* =29.5 kg mol^−1^). The same stategy was also successful for the stereoselective ROCOP of cyclopentene oxide or 3,4‐epoxytetrahydrofuran, which both formed highly isotactic polymers (*T_m_* =141 and 232 °C, respectively).

Organocatalysts for COS/(PO/CHO/PGE) ROCOP include Et_3_B/Lewis bases which showed lower activity, but slightly higher TH selectivity (TOF 61–119 h^−1^, 25 °C, 0.1 mol %, *M_n_* =14.4–92.5 kg mol^−1^) than Cr^III^‐salen catalysts.[^166^, ^167^, ^168^] Bifunctional Lewis bases, such as *N*,*N*,*N′*,*N′*‐tetraethylethylenediamine, with Et_3_B showed higher activity (TOF 8000–22 500 h^−1^, 0–80 °C, 0.1 mol %, *M_n_* =46.6–81.0 kg mol^−1^) and maintained high selectivity for polymer at 80 °C. A mechanistic switch from ROCOP to ROP was achieved, whereby COS/PO ROCOP was followed by PO homopolymerisation to form block polymers. Thiourea/Lewis base (/alcohol) systems were effective in COS/PO ROCOP (TOF 10–112 h^−1^, 25 °C, 0.05–0.1 mol %, *M_n_* =11.3–98.4 kg mol^−1^), which is surprising given that the same systems fail with CO_2_ ROCOP.^[169]^

**Figure 24:**
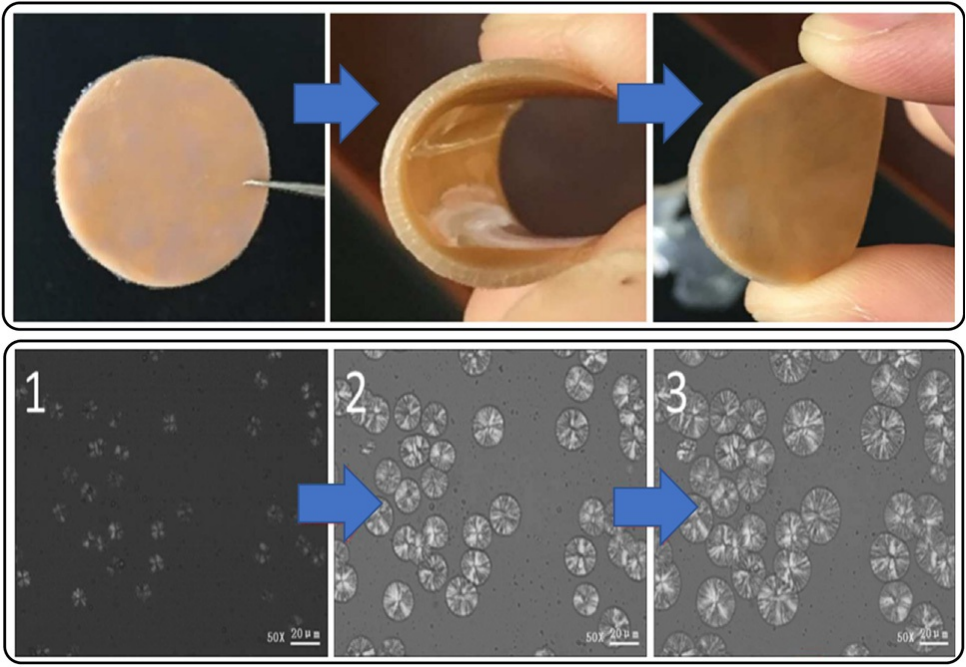
Top: Elastomeric COS/oxetane copolymer wafer. Bottom: Polarized light microscopy image of the crystal growth of the molten COS/oxetane copolymer at 96 °C.^[170]^ Copyright 2016 American Chemical society.

Oxetane/COS ROCOP using Cr^III^‐salen/PPNCl (TOF 4–62 h^−1^, 40–130 °C, 0.4 mol %, *M_n_* =4.9–40.9 kg mol^−1^) or Et_3_B/PPNCl catalyst systems both yield perfectly alternating polymers, without O/S scrambling.[^170^, ^171^] Minor amounts of the cyclic by‐product thio‐c6 c formed at the end of the polymerization, which suggests that the reactions occurred by direct COS/oxetane ROCOP rather than thio‐c6 c ROP. The polymers show high crystallinity (*T_m_* =128 °C, *M_n_* =30 kg mol^−1^), which contrasts with the amorphous all‐oxygen analogue PTC (*T_g_* =−20 °C). Furthermore, the *T*_m_ values of related aliphatic polycabonates (180–275 °C) are close to their decomposition temperatures.[^172^, ^173^, ^174^, ^175^] In contrast, the thiocarbonate from COS/oxetane ROCOP showed *T_m_* =128 °C and *T_d_* =229 °C, thus giving a reasonable processing temperature range. This allowed for hot‐press processing to produce an elastomeric material (Figure 24), although its tensile properties remain to be reported.

## 10 ROCOP of CS_2_ with Epoxides, Oxetane, and Thiirane

**Figure 25:**
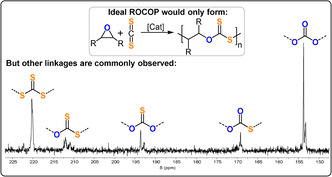
CS_2_/epoxide ROCOP and ^13^C{^1^H} NMR spectrum illustrating the polymer functionalities accessible when O/S scrambling occurs.

CS_2_/PO ROCOP was first reported in the 1970s by Adachi et al., who used a mixture of Et_2_Zn and HMPA (TOF <1 h^−1^, 25 °C, *M_n_* =0.6 kg mol^−1^).^[176]^ Two key findings resulted: a) O/S scrambling formed ‐S‐C(=S)‐O‐ linkages as well as other linkages (Figure 25); b) the CS_2_/epoxide system dramatically influenced the catalytic activity and selectivity. This latter observation is different from CO_2_/epoxide ROCOP, where the catalytic performance is commonly independent of CO_2_ pressure.

Heterogeneous DMC catalysts for CS_2_/PO ROCOP yielded polymers with low molecular weights and with moderate/good activities (52–182 g g^−1^ h, *M_n_* =1.2–5.4 kg mol^−1^).^[177]^ The resulting polymers were oxygen‐enriched (ca. 35 % theoretical max. S) and exhibited nearly all the possible permutations of linkages. Moreover, carbonyl sulfide (COS) was detected in increasing quantities as the reaction progressed. This was attributed to COS being more easily, or perhaps rapidly, incorporated, thus rationalizing the observation that ‐S‐C(=O)‐O‐ linkages were the most prevalent.

Darensbourg et al. reported that Cr^III^‐salen/PPNCl showed a better catalytic performance in CS_2_/CHO ROCOP, but again linkage scrambling occurred (TOF 124 h^−1^, 50 °C, 0.08 mol %, *M_n_* =26.5 kg mol^−1^).^[178]^ All linkages formed simultaneously in the polymers, which indicates that linkage exchange was faster than propagation. The resulting polymer was semi‐crystalline (*T_g_* =122 °C; *T_c_* =152 °C, *T_d_* =220 °C), in contrast to amorphous PCHC (*T_g_* =110–130 °C). The crystallinity was reinforced by weak attractive interchain S⋅⋅⋅S interactions. CS_2_/CPO ROCOP was also feasible using this catalyst (TOF 146 h^−1^, 80 °C, 0.067 mol %), even though the same catalyst produces only c5c in the case of CO_2_/CPO reactions.[^179^, ^180^]

CS_2_/oxetane ROCOP using a Cr^III^‐salen/PPNCl catalyst yielded polymers with high sulfur contents (TOF 83 h^−1^, 80 °C, 0.1 mol %, *M_n_* =13.7 kg mol^−1^, >95 % max. S).^[181]^ Increasing the CS_2_ concentration resulted in both the molar mass and the desired ‐S‐(C=S)‐O‐ linkage selectivity increasing. Linkage selectivity decreased as the temperature was increased, which suggests that scrambling is entropically favoured. The presence/absence of co‐catalyst also influenced the extent of scrambling, which suggests some exchange reactions do not involve the metal.

Clearly, controlling the O/S scrambling side reactions is a significant challenge in CS_2_/epoxide ROCOP. Understanding pathways that form particular linkages remains more hypothetical than proven; nonetheless, if it were possible to control these processes in the future it might be feasible to produce polymers featuring (ABC)_*n*_ or (ABCD)_*n*_ monomer sequences instead of the expected, and more common, (AB)_*n*_.

**Figure 26:**
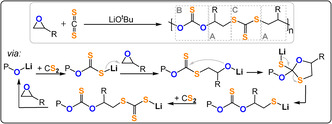
CS_2_/epoxide ROCOP, catalysed by LiO^*t*^Bu, to produce (ABAC)_*n*_ copolymer; R=Me, Et.^[182]^

**Figure 27:**
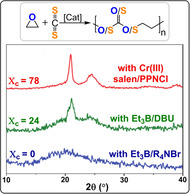
XRD data for CS_2_/EO copolymers from bicomponent catalysts show different degrees of crystallinity.^[184]^ Copyright 2020 Wiley.

In 2016 Werner and co‐workers realized an (ABAC)_*n*_ copolymer, with 92 % sequence selectivity, from CS_2_ with PO or butylene oxide and LiO^*t*^Bu as the catalyst (TOF 50 h^−1^, 25 °C, 0.125 mol %, *M_n_* =132 kg mol^−1^).^[182]^ The catalyst leads to chains with a somewhat unusual HH‐TT selectivity and a tentative mechanism was proposed (Figure 26). Accordingly, the propagating alkoxide may attack an adjacent ‐O‐(C=S)‐S‐ linkage to afford a ‐O‐(C=S)‐O‐ linkage with concomitant formation of a thiolate chain end. The same lithium alkoxide catalyst also produced an isotactic polymer from CS_2_/*R*‐PO ROCOP which has a higher *T_g_* value than the atactic form (*T_g_* =30 °C for isotactic vs. 13 °C for atactic). Organocatalysts composed of Et_3_B/lewis base were also effective for CS_2_/PGE ROCOP and afforded only trithio‐ or monothiocarbonate linkages, although with worse overall performance than metal systems.^[183]^ CS_2_/EO ROCOP using bicomponent Et_3_B or Cr^III^‐salen catalysts revealed that the extent of the O/S scrambling changed the material properties from completely amorphous (*T_g_* =−18.6 to −35.1 °C) to highly crystalline (*T_g_* ≈−34 °C, *T_m_* =118–211 °C; Figure 27).

Whereas sulfur‐rich segments tend to be crystalline, the all‐oxygen carbonate linkages form amorphous regions. Another interesting feature of these copolymers is their ability to be degraded by oxidants. The immersion of solid polymer in 30 % H_2_O_2_ for 12 h resulted in degradation to oligomers (*M_w_* =1.8 kg mol^−1^) with formation of sulfones (R_2_SO_2_) and sulfonic acids (RSO_3_H).^[184]^

**Figure 28:**
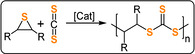
CS_2_/thiirane ROCOP forming poly(trithiocarbonate).

Early investigations of CS_2_/ES and PS ROCOP used highly toxic CdEt_2_ or Hg(SBu)_2_ catalysts—or metal‐phenolate catalysts in the case of CO_2_/PS ROCO—and formed large quantities of thioether as well as heterocarbonate links.[^185^, ^186^, ^187^] Nozaki and co‐workers reported CS_2_/PS ROCOP using a Cr^III^‐salen/PPNCl catalyst, which produced highly alternating trithio‐PPC with high polymer selectivity (92 %) and good activity (TOF 76 h^−1^, 25 °C, 0.2 mol %, *M_n_* =44.6 kg mol^−1^, *T_g_* =25 °C, *T_d_* >200 °C; Figure 28).^[188]^ The poly(trithiocarbonate) was only sparingly soluble in common organic solvents but was highly soluble in CS_2_, thereby highlighting future processing challenges for highly heteroatom‐rich polymers. The polymers showed high refractive indices (RI=1.78 for CS_2_/PS, 1.73 for CHS/PS; in comparison, the RI values of PPC is 1.46 and PCHC is 1.48). In general, high RI polymers are investigated for their optoelectronic applications, such as for lenses in image sensors, optical layers in LCD displays, encapsulants for LEDs, and anti‐reflection coatings.[^154^, ^189^] Compared to alternative inorganic compounds, the ROCOP polymers may benefit from mechanical flexibility, impact strength, processability by moulding or casting, have low molecular weights, and potentially have lower costs. Although S‐containing ROCOP polymers have not been explored for such applications, the broad monomer scope and potential for terpolymerisation may allow future optimisation of the properties.

Both CS_2_/PS and CS_2_/CHS copolymers exhibited antimicrobial activity against *Escherichia coli* and *Staphylococcus aureus*.^[190]^ Bacterial cultures in contact with polymer films were assessed for their cell viability by counting surviving colonies. Although poly(cyclohexene trithiocarbonate) was more effective against *E. coli* (20 % cell viability for CS_2_/PS and >10 % for CS_2_/CHS after 24 h contact time), the biocidal performance was reversed for *S. aureus* (25 % cell viability for CS_2_/PS and 50 % for CS_2_/CHS after 24 h contact time).

## 11 ROCOP of Thioanhydrides with Epoxides or Thiiranes

Very recently, the sulfur analogues of anhydride/epoxide ROCOP were reported to deliver poly(thioester) structures inaccessible by conventional routes.[^191^, ^192^, ^193^]

**Figure 29:**
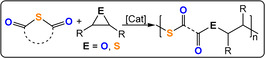
ROCOP of thioanhydride with epoxides or thiiranes.

**Figure 30:**
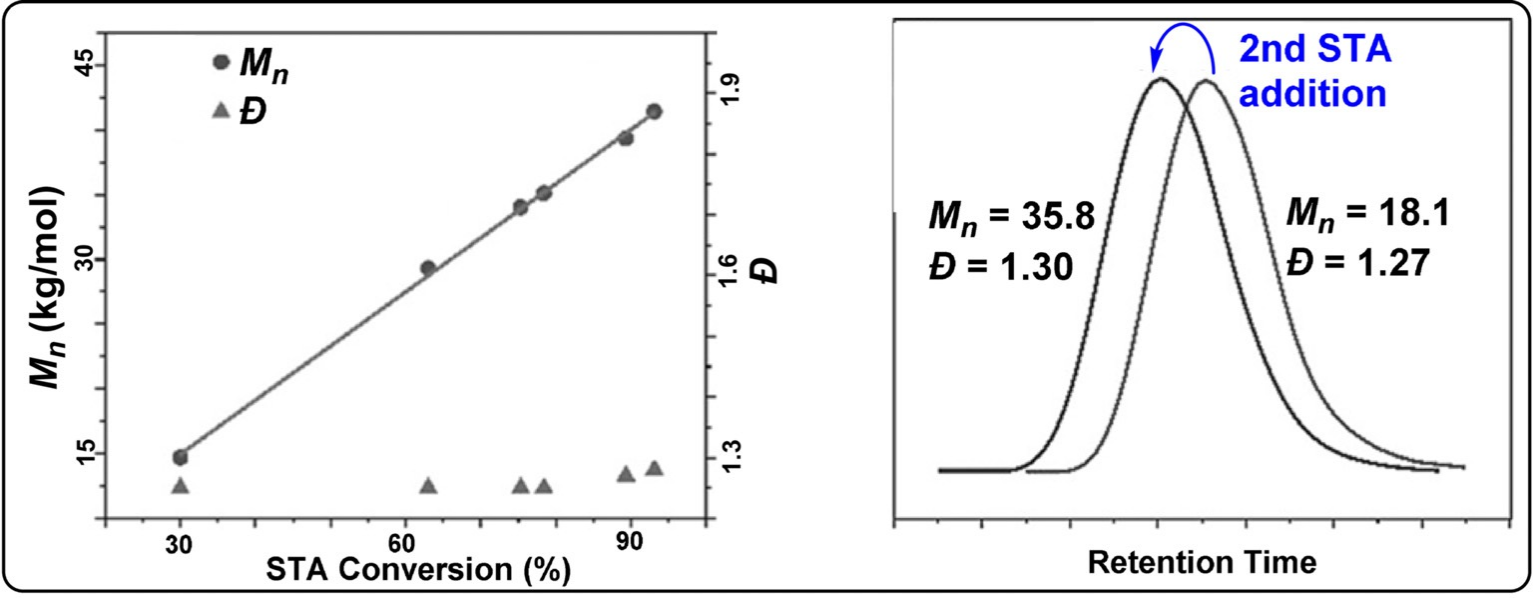
PPNOAc‐catalysed STA/PS ROCOP. Left: Controlled molecular weight increase versus conversion.^[191]^ Right: GPC traces before and after second monomer addition. Copyright 2020 Wiley.

Lu and co‐workers reported ROCOP of thiiranes with stearic (STA) or glutaric thioanhydride (GTA) using Lewis base/PPNX catalysts (Figure 29).^[191]^ The most effective was PPNOAc, which delivered polymers quickly with molar masses close to theoretical expectations (TOF 787 h^−1^, 25 °C, 0.4 mol %, *M_n_* =53.6 kg mol^−1^, for STA/PS; Figure 30).

Polymerisation control was good, and block polymers were prepared by the addition of a second thioanhydride after consumption of the first. The copolymer showed a high refractive (RI=1.79) and moderate melting temperature (*T_m_* =80 °C), with the latter value increasing to 90 °C when *R*‐PS was used. The onset of thermal decomposition occurred at 230–300 °C (*T_g_* =−22–60 °C) depending on the thiirane employed, which suggests the materials have a reasonable processing window.

Phthalic thioanhydride (PTA)/PO ROCOP using Cr^III^‐salen/PPNCl catalysts showed good performance (TOF 30–1420 h^−1^, 25–100 °C, 0.1–0.001 mol %, *M_n_* =18.2–60.1 kg mol^−1^).^[192]^ The polymer contained thioester, ester, and thioether linkages, was regio‐random, and lacked long‐range order. The linkage scrambling was attributed to intramolecular and/or intermolecular transesterification reactions akin to CS_2_/epoxide ROCOP. Scrambling increased with temperature and influenced the polymer's glass transition temperature (*T_g_* =62 °C from reactions at 100 °C, *T_g_* =70 °C from reactions at 25 °C, both with *M_n_* ≈18 kg mol^−1^). A tethered Lewis base/Cr^III^‐salen catalyst showed fewer transesterifications (TOF 94–567 h ^−1^, 0.1–0.01 mol %, 25–70 °C, *M_n_* =15.9–49.7 kg mol^−1^).^[193]^ Highly regioregular poly(ester‐*alt*‐thioester) formed at 25 °C but, once again, higher temperatures resulted in scrambling. This catalyst also successfully copolymerized thioanhydrides with other epoxides to afford amorphous polymers with glass transition temperatures from 35 to 161 °C, although with low molar masses (*M_n_* =5.7–17.5 kg mol^−1^). Some poly(thioesters) are biosynthesised by *E. coli* which suggests alternative metabolic pathways based on sulfur reactions that might be relevant to biological compatibility and biodegradation.^[194]^

## 12 ROCOP of S_8_ with Thiiranes

The ring‐opening polymerisation of elemental sulfur (S_8_) is thermodynamically disfavoured at temperatures below 159 °C but occurs readily at higher temperatures.^[195]^ This unusual phenomenon arises because S_8_ ROP is entropically favoured, that is, Δ*S*(polymerisation)>0.^[196]^ Elemental sulfur is a waste product of petrochemical refining, with 70 Mt produced annually, most of it by hydrodesulfurisation.^[197]^ Although a significant portion is used to make sulfuric acid, rubber, and fertilizer, excess sulfur accumulates in large over‐ground storage facilities.

**Figure 31:**
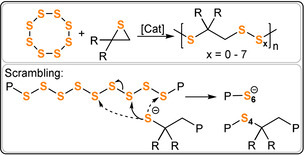
S_8_/thiirane ROCOP and linkage scrambling to form polysulfides (S_x_).

Penczek and Duda demonstrated S_8_/thiirane ROCOP (ES, PS, 2‐Me‐PS) using sodium thiophenolate crown ether or CdCO_3_ catalysts (Figure 31).[^198^, ^199^, ^200^, ^201^, ^202^] Altering the S_8_/thiirane ratio from 1:1 to 10:1 modulates the sulfur uptake and reaches a maximum at 85 wt % (with PS). ^13^C NMR spectroscopy shows a range of polysulfide (S_x_) linkages, and their formation was attributed to a linkage scrambling process akin to the transesterification reactions discussed earlier.

Analysis of the initial rates demonstrated that the sulfide chain end attacked S_8_ about 10 times faster than thiirane. The ‐S_9_^−^ intermediate attacked thiirane about 100 times faster than it formed S_8_. Curiously, the anionic polysulfide chain ends became less reactive as the preceding S_*n*_ group gets longer, thus suggesting some negative charge delocalisation along the chain. Similar to other ROCOPs, small cyclic by‐products were formed, including cyclic tri‐, tetra‐, and pentasulfides. These cyclic compounds reached a maximum concentration and then decreased over time, thus suggesting they undergo ring‐opening polymerization; this was later confirmed by homo‐ and copolymerisation with S_8_.^[201]^ Sulfur/heterocycle ROCOP reactions yielded polymers which are amorphous elastomers (*M_n_* =10–100 kg mol^−1^). In contrast to polysulfides S_*n*_, the polymers were stable with respect to depolymerisation as well as to extrusion of sulfur under ambient conditions. Films cast from polymer solutions retained transparency after storage for 4 years. Poly(styrene‐*alt*‐sulfur), obtained from S_8_/styrene sulfide ROCOP, showed that increasing the sulfur content (although not quantified) decreased the *T_g_* value from 58 to 43 °C, which matched previous reports that increasing the wt % of sulfur reduced the brittleness.^[203]^

It is emphasised that sulfur–sulfur bonds are often dynamic, a property exploited in autonomously self‐healing polyurethane elastomers, where macroscopic damage was healed through dynamic disulfide bonds.^[204]^ By simply bringing two ends of the cut specimen bar into contact, the mechanical properties were nearly completely restored after 24 h. In the future, these ROCOP polymers could be explored as self‐healing materials.

## 13 ROCOP of SO_2_ with Epoxides

Sometimes ROCOP occurs spontaneously because the two monomers (A and B) first form an activated monomer adduct (A‐B), which then forms chains by step growth or other mechanisms.

**Figure 32:**
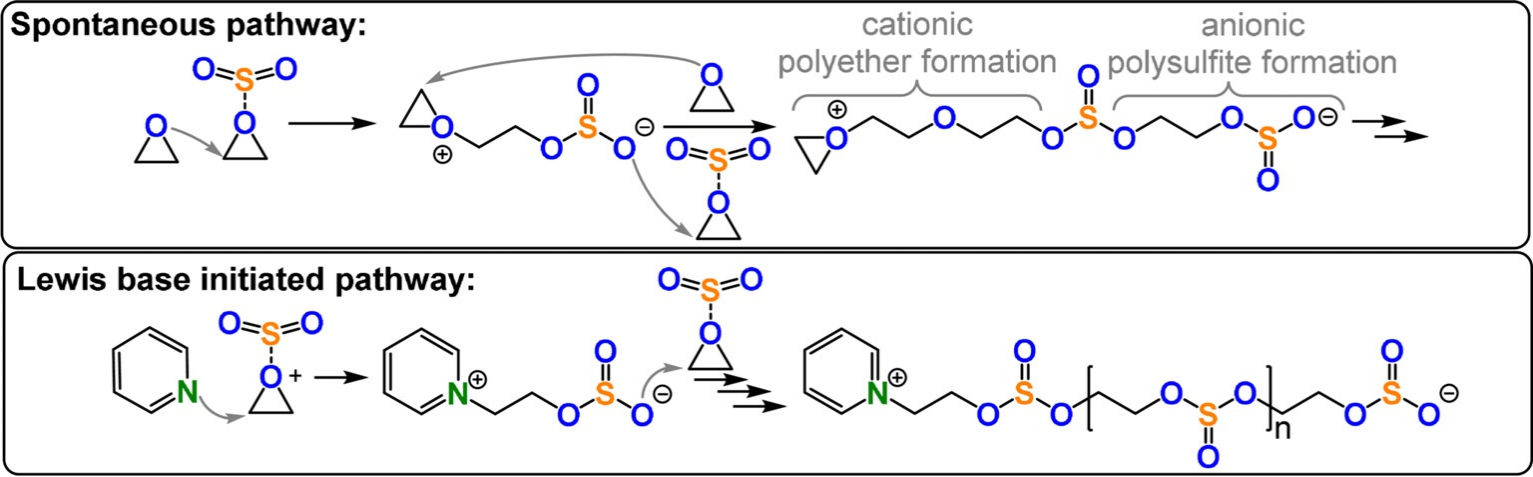
Base‐initiated SO_2_/epoxide ROCOP versus spontaneous copolymerisation.

**Figure 33:**
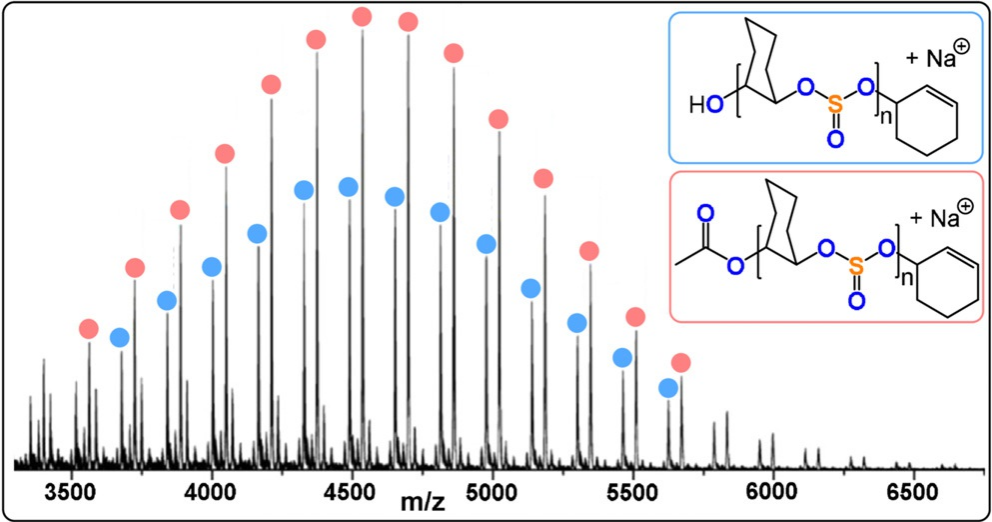
MALDI‐TOF MS spectrum of a SO_2_/CHO copolymer showing chains initiated from trace amounts of H_2_O.^[210]^ Copyright 2020 American Chemical Society.

Such an activated monomer mechanism was reported for SO_2_/epoxide ROCOP, which formed low molar mass polymers enriched with ether linkages (Figure 32).[^205^, ^206^, ^207^, ^208^, ^209^] Higher molar masses, sulfate incorporation, and reaction rates were achieved using Lewis base initiators such as N‐heterocycles, phosphines, or salts (TOF 20–35 h ^−1^_,_ 50 °C, 0.1–1 mol % pyridine, *M_n_* =11.9–14.2 kg mol^−1^). Initiation occurred from zwitterions (LB^+^CR_2_CR_2_OSO_2_^−^) and the activity was dependent on the ionization of the initiator. Propagation occurs when SO_2_ activates the epoxide and, in general, the rates depend on the epoxide, the SO_2_, and Lewis base. Cyclic and linear polymers form, which limits the molar masses obtained (e.g. SO_2_/EO ROCOP=9.2–14.2 kg mol^−1^). Using poly(vinylpyridine) as an initiator allows separation of the linear and cyclic polymers which are bound to the macro‐initiator. Thermal decomposition of the SO_2_/EO copolymer, at relatively low temperature (*T_d_* =216 °C), led to mixtures of cyclic five‐membered sulfites and EO, which were recovered in about 44 % yield. The depolymerisation was catalysed by Brønsted or Lewis acids, which reduced the degradation temperature to 50 °C, and was also accelerated by UV irradiation. Lu and co‐workers reported PPNX‐intitated (X=OAc, Cl) SO_2_ ROCOP with a range of epoxides (TOF 13–1116 h^−1^, 25–50 °C, 0.2 mol %, *M_n_* =3.1–19.8, 88–99 % sulfite content).^[210]^ The authors noted that chain transfer reactions with trace ammouts of water limit the molecular mass of the linear polymers (Figure 33).

The use of zinc glutarate for SO_2_/PO ROCOP yielded polymers with molar masses up to 42 kg mol^−1^ and 90 % sulfite contents (*T_g_* =14 °C).^[211]^ Using a Cr^III^‐salen catalyst without a co‐catalyst for SO_2_/CHO ROCOP gave higher molar masses and sulfite contents compared to the spontaneous pathway (TOF 80 h ^−1^, 60–90 °C, 0.1 mol %, *M_n_* =9.3–27.7 kg mol^−1^, *T_g_* =53 °C, *T_d_* =205 °C, 64–77 % sulfite).^[212]^ Similar catalysts immobilized on cellulose supports enabled catalyst recycling up to three times with little loss of performance.^[213]^ Cr^III^‐salen/PPNCl catalysts allow random terpolymerisation of CO_2_, SO_2_, and CHO, which may be relevant to some petrochemical waste streams where CO_2_ is contaminated with SO_2_.^[214]^ The terpolymers were enriched with sulfite, thus reflecting the higher electrophilicity of SO_2_ compared to CO_2_.

## 14 ROCOP of RNCO with Epoxides and RNCS with Thiiranes

**Figure 34:**
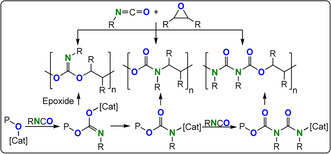
RNCO/epoxide ROCOP leading to multiple different linkages.

Early studies demonstrated that slow alternating ArNCO/EO ROCOP was possible when employing a AlEt_3_/H_2_O (2:1) catalyst system (TOF 0.3 h^−1^, 25 °C for PhNCO).[^215^, ^216^] The addition of the isocyanate to the epoxide occurred with retention of the C=N bond to form an acetal linkage or with retention of the C=O bond to form a urethane linkage (Figure 34); the semi‐crystalline polymer contained two thirds acetal linkages (*M_n_* =1–2.1 kg mol^−1^, *T_m_* =80–83 °C). In comparison, an isomeric polymer synthesized by ROP and that only contains urethane linkages showed a *T_m_* =192 °C. These findings emphasize the importance of linkage connectivity in moderating material properties. It should be noted that alternating polyurethanes, such as those formed through ROCOP, are totally different to the polyurethanes currently produced through the condensation of polyols and di‐isocyanates.^[7]^ These commercial products contain a much lower wt % of urethane linkages and combine hard and soft domains which are integral to their properties.

Faster and more selective ArNCO/CHO ROCOP (TOF >1000 h^−1^, 80 °C, 0.2 mol %, *M_n_* =3–8 kg mol^−1^) was achieved using a Mg^II^_2_ catalyst [LMg_2_OAc_2_], but curiously the di‐Zn^II^ and di‐Co^III^ derivatives as well as the Co^III^‐ and Cr^III^‐salen/PPNCl catalysts were completely inactive.^[217]^ Depending on the conditions, the polymer contained either mostly urethane, RO‐(C=O)‐NR_2_ or allophanate (RO‐(C=O)‐NR‐(C=O)‐NR_2_) linkages (with the latter formed by two consecutive ArNCO insertions). The polymers decomposed (*T_d_* =180–210 °C) before any phase transitions could occur, with the exception of the *p*‐fluoro‐substituted polyurethane with *T_g_* =181 °C.

The polymerisation of PO, EO, octene oxide, or allyl glycidyl ether with tosyl isocyanate (TsNCO) in the presence of Et_3_B/(PPN/R_4_N)Cl catalysts resulted in polyurethane materials with moderate–high molar masses (TOF 10 000 h^−1^, 25 °C, 1–0.1 mol %, *M_n_* =15–225 kg mol^−1^).^[218]^ Using monofunctional chloride initiators yielded polyurethanes showing monomodal molar mass distributions, whilst phosphazene and diol catalysts yielded the more useful telechelic polyurethanes. The TsNCO/PO copolymer was amorphous with *T_g_* =107 °C (*M_n_* =20 kg mol^−1^, *T_d_* =242 °C), which is significantly higher than that of the related polycarbonate PPC (*T_g_* =22–40 °C, *T_d_* ≈242 °C). By changing the epoxide, it was feasible to control *T_g_* in the resulting polyurethanes to give values as low as −24 °C; it was also noted that TsNCO/EO yields a semi‐crystalline polyurethane, with *T_m_* =61 °C (*T_c_* =44 °C). In related study, Oct_4_NBr/(*i*Bu)_3_Al was investigated for ArNCO/butylene oxide ROCOP, but gave poor polymer and linkage selectivity.^[219]^

**Figure 35:**
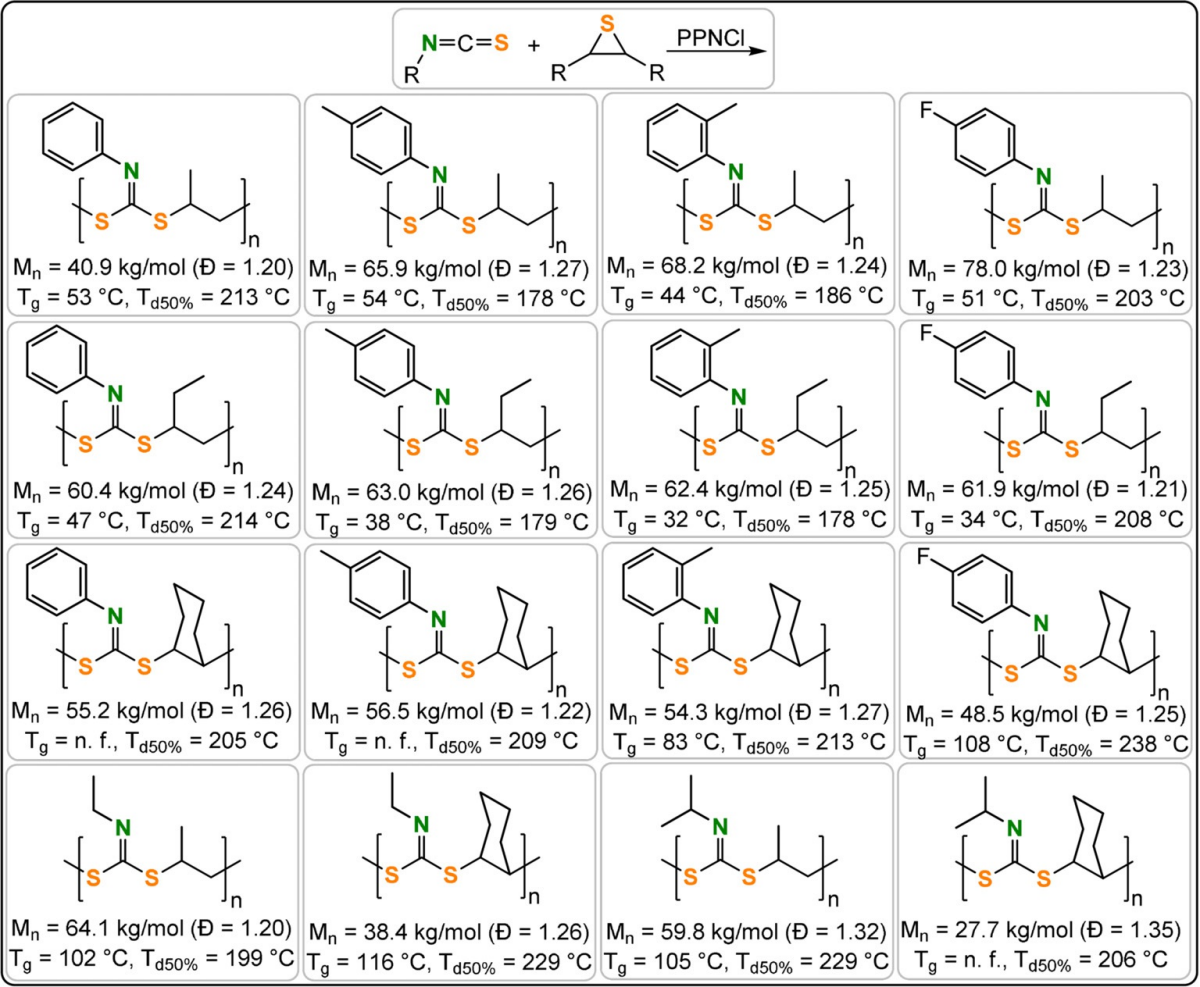
Polymers formed from RNCS/thiirane ROCOP with thermal properties.^[221]^

Using ^*n*^BuLi as the initiator resulted in successful RNCS/ES ROCOP to produce semi‐crystalline poly(imino dithioacetal), [‐CH_2_CH_2_‐S‐C(=NR)‐S‐]_*n*_ (*T_m_* =63–128 °C depending on R; *M_n_* =25–60 kg mol^−1^).^[220]^ Polymerisation rates were increased when using coordinating solvents, for example, THF or (Me_2_N)_3_P=O, which enhance the nucleophilicity of the dithiocarbamate chain end through its coordination to lithium. The terpolymerisation of different RNCS species with ES followed the reactivity ratio trends, with aryl‐NCS being more reactive than alkyl‐NCS. For example, PhNCS/EtNCS/ES ROCOP selectively formed the PhNCS/ES block first followed by the EtNCS/ES block. When ES was used in excess it was also consumed by ROP, thereby forming a final poly(thioether) block, together with slow degradation of the polymer. The RS‐(C=NPh)‐SR repeat units can be hydrolysed upon contact with dilute aqueous acids, thereby resulting in the release of aniline (PhNH_2_) and formation of semi‐crystalline polymers with RS‐(C=O)‐SR linkages which are much less soluble than the starting polymer. Wu and co‐workers reported strictly alternating RNCS/thiirane ROCOP, by using PPNCl initiators, and achieved good to excellent activity and molar masses (TOF 21–1162 h^−1^, 0.4–0.1 mol %, 25–100 °C, *M_n_* =19.9–142 kg mol^−1^).^[221]^ The different monomer substitution patterns highlight the wide range of structures accessible with this ROCOP method (Figure 35).

## 15 ROCOP Involving Aziridines

**Figure 36:**
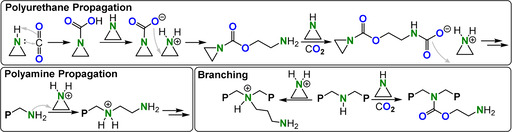
Spontaneous CO_2_ and EI ROCOP.

Aziridines are more nucleophilic than epoxides or thiiranes by virtue of the lone pair of electrons on the N atom. Hence, they form stable carbamate adducts with carbon dioxide, effectively the reverse of the reactivity with SO_2_, where adduct formation resulted from its increased electrophilicity. For example, ethyl aziridine (EI) reacted with CO_2_ at −27 °C to form the salt [EICO_2_]^−^[EIH]^+^. Upon heating this salt in mixtures of EI and CO_2_, the anion [EICO_2_]^−^ attacked and ring‐opened the activated aziridine cation [EIH]^+^ to effect copolymerisation by an activated monomer mechanism (Figure 36).[^222^, ^223^] Two competitive polymerisation pathways were observed in parallel: 1) Formation of polyamine linkages by cationic aziridine ROP and 2) branching from these amine links.

Ikeda and co‐workers first described catalyst‐free CO_2_/EI ROCOP to obtain polymers with low urethane contents (<31 mol %), and using propylene imine (PI) resulted in polymers showing up to 70 % urethane linkages.[^222^, ^223^] The potential for these monomers to undergo spontaneous ROCOP did not rule out the need and possible benefits for catalysed pathways.^[224]^ For example, CO_2_/N‐Ph‐EI ROCOP using a metal catalyst resulted in higher polymer yields, urethane content, and molar masses compared to uncatalyzed controls; the best catalysts were MnCl_2_(H_2_O)_4_ and Mn(acac)_2_. Catalyst systems comprising mixtures of Et_2_Zn with polyphenols/phenylamines formed perfectly alternating oligourethanes (*M_n_* <0.6 kg mol^−1^).^[225]^ Similarly, a ternary Y(Cl_3_CCO_2_)_3_/ZnEt_2_/glycerine catalyst for CO_2_/PI ROCOP resulted in polymers with 80 % urethane linkages (70 °C, 40 bar, *M_n_* ≤30 kg mol^−1^).^[226]^

**Figure 37:**
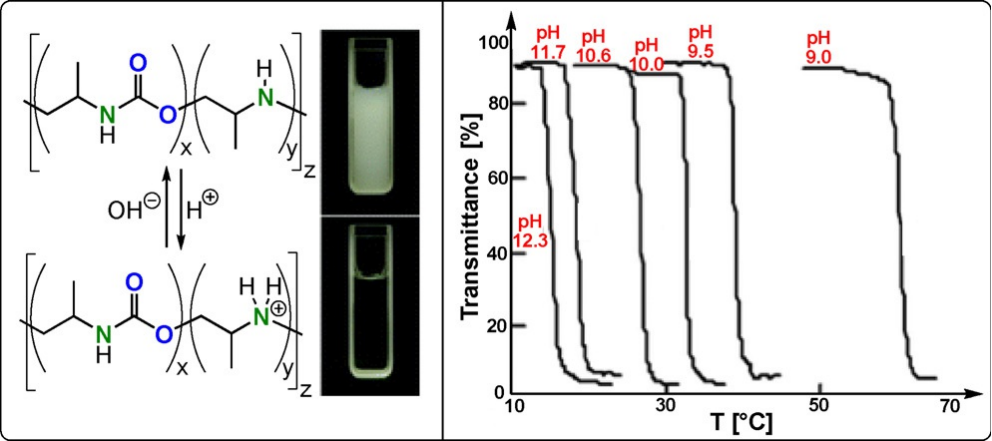
Aziridine/CO_2_ ROCOP data. Left: pH‐dependent water solubility of poly(urethane‐*ran*‐amine). Right: Temperature‐dependent light transmittances of aqueous poly(urethane‐*ran*‐amine) solutions with pH.^[229]^ Copyright 2005 RSC.

Ikariya and co‐workers investigated the use of supercritical CO_2_ (up to 220 bar) for CO_2_/PI ROCOP.[^227^, ^228^] Whereas the aziridines were soluble in supercritical CO_2,_ the polyurethanes precipitated from it, presumably as a consequence of intermolecular hydrogen bonding (Figure 37). Using dimethylacetamide as a co‐solvent prevented precipitation and homogenized the mixture, thereby allowing the isolation of polymers with higher molar masses (*M_n_* =210 kg mol^−1^, 74 % urethane linkages).

The polymers contain hydrophilic polyamine and hydrophobic polyurethane sequences in various proportions and so, depending on the reaction conditions (CO_2_ pressure, solvent, etc), they show different lower critical solution temperatures (LCST) in water. The LCST arises from an unfavourable entropy of mixing between the polymer and solvent and is observed for other polymers, with a common example being poly(*N*‐isopropylacrylamide).^[230]^ In the case of these CO_2_/aziridine copolymers, the amine linkages are susceptible to protonation/deprotonation and thus the solution pH also controls the LCST: under acidic conditions there is no LCST and its value decreases as the pH increases.^[229]^

**Figure 38:**
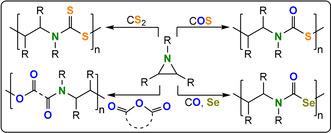
ROCOP between aziridines and CS_2_, COS, COSe, or cyclic anhydrides.

Ikeda and co‐workers reported that water‐catalysed CS_2_/*N*‐cyanoethyl‐EI ROCOP formed a poly(dithiocarbamate) which was semi‐crystalline (*T_m_* =155 °C) and poorly soluble in all solvents, except for DMSO (Figure 38).^[231]^ The rate law showed dependencies of: [N‐EtCN‐EI]^2^[CS_2_][H_2_O] and was interpreted by a rate‐determining step involving attack by an N‐C(=S)‐S^−^ group from an activated monomer complex on a protonated aziridine (protonation from water). The CS_2_/aziridine copolymer showed high alternation, irrespective of the starting quantities of each monomer, thus suggesting that the aziridine ROP was significantly less favourable compared to other CO_2_/aziridine ROCOPs.

**Figure 39:**
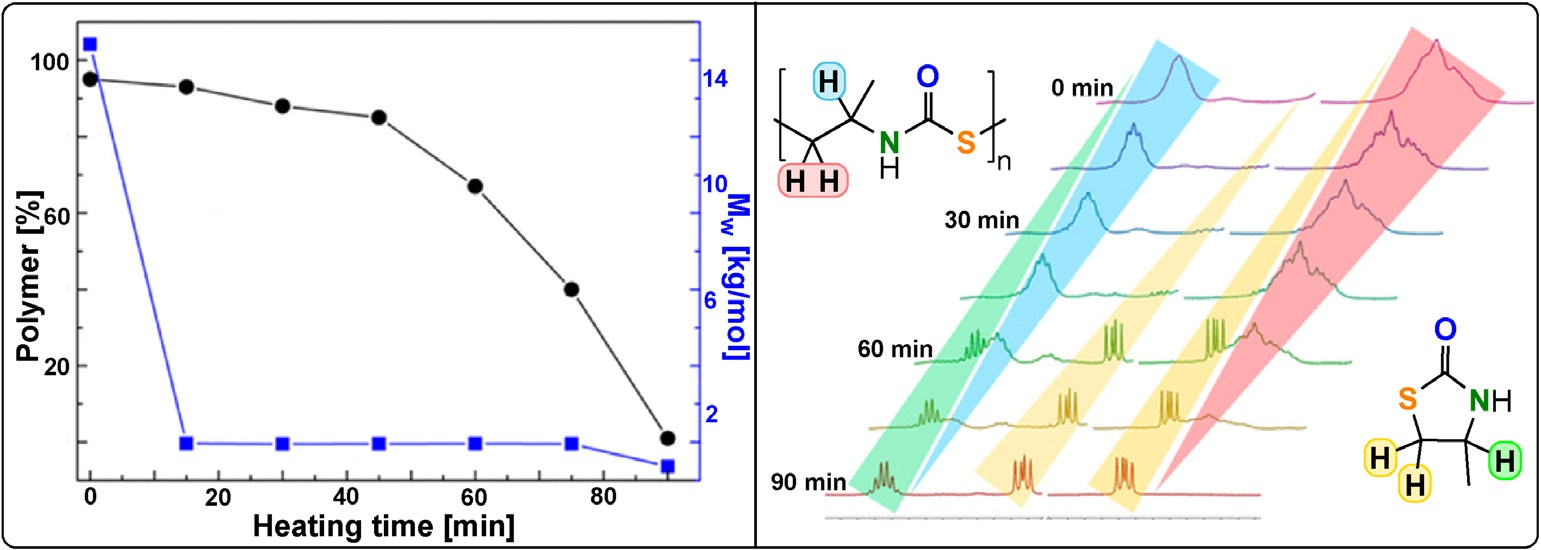
Thermally induced depolymerisation of neat COS/PI copolymer at 200 °C as monitored by GPC and NMR spectroscopy.[^232^, ^233^] Copyright 2020 American Chemical Society.

COS/aziridine ROCOP is also spontaneous through an activated monomer mechanism.^[234]^ Using COS pressures of 10 to 20 MPa results in the formation of polymers with molar masses up to 15 kg mol^−1^ within 2 hours at room temperature.[^232^, ^233^] The highly alternating polymers were macrocyclic, as determined by MALDI‐TOF mass spectrometry, and were cleanly depolymerized at 200 °C into the five‐membered cyclic thiourethane. These polymers were shown to effectively absorb heavy metal salts, such as HgCl_2_ and PbCl_2_, from aqueous solutions. The absorbed metal salts were isolated after thermal depolymerisation and removal of the small molecule cyclic compounds by distillation (Figure 39). Whereas the COS/PI copolymer was amorphous (*T_g_* =90 °C), the COS/(*N*‐ethyl‐EI) (*T_m_* =170 °C) and *N*‐butyl‐Az (*T_m_* =137 °C) copolymers are semi‐crystalline. There is also a tentative precedence for spontaneous COSe/aziridine copolymerisation.^[235]^

Poly(ester‐amide)s, the formal products of anhydride/aziridine ROCOP, combine the biodegradability of polyesters with the thermal and mechanical properties of polyamides—these desirable features have resulted in applications ranging from drug delivery systems to hydrogels, composite matrices, and tissue engineering scaffolds.^[236]^ In some cases, anhydride/aziridine ROCOP was spontaneous, but was characterized by poor control over the molar mass and amine linkages.[^237^, ^238^] Quantitative monomer alternation was achieved with Lewis base/alcohol organocatalysts. Cyclic polymers were isolated in some cases when mono‐/bicyclic anhydrides and *N*‐benzyl‐substituted aziridines were applied (TOF 1–10 h^−1^, 70 °C, 0.2‐1 mol %, *M_n_* =4.4–34.1 kg mol^−1^, *T_g_* =41–126 °C, *T_d_* =258–311 °C).^[239]^ As with cyclic polymers formed by macrocyclisation during termination, applying higher BnOH loadings resulted in more linear chains.

**Figure 40:**
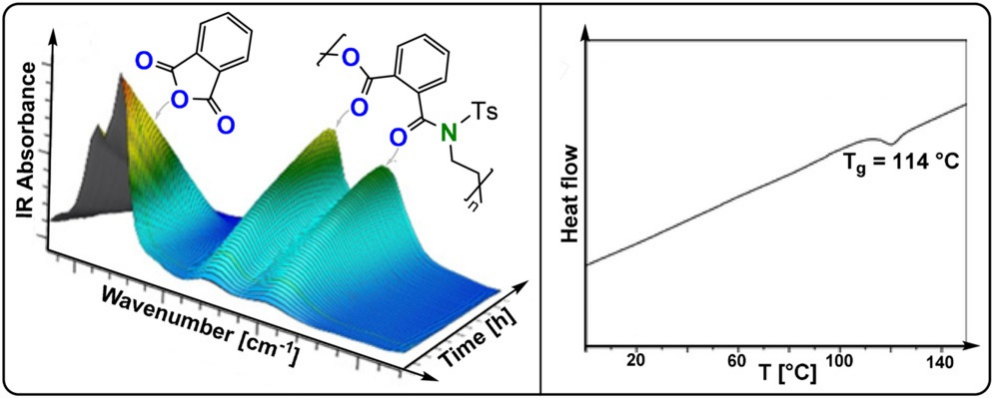
Anhydride/aziridine ROCOP data. Left: In situ FTIR spectroscopic analysis of PA/*N*‐Ts‐EI ROCOP.^[240]^ Right: DSC thermogram of the copolymer. Copyright 2020 Wiley.

Good molar mass control was achieved for PA/*N*‐tosyl‐EI ROCOP (TOF 14–27 h^−1^, 50 °C, 0.3‐2 mol %, *M_n_* =5.1–35.7 kg mol^−1^, *T_g_* =114 °C, *T_d_* =265 °C).^[240]^ The authors rationalised the better control through suppression of transacylation side reactions arising from the electron‐withdrawing tosyl substituent, which increased the selectivity for linear polymers. Compared to the closest epoxide/anhydride ROCOP analogue poly(propylene oxide‐*alt*‐PA) (*T_d_* =269 °C, *T_g_* =55 °C), the incorporation of amide linkages increases the *T_g_* (Figure 40). When excess aziridine was employed, a mechanistic switch to *N*‐Ts‐EI ROP was feasible, with formation of a polyamine block after the poly(ester amide) block. The finding that homogeneous organocatalysts greatly improve control in anhydride/aziridine ROCOP might imply that similar catalysts could achieve living heteroallene/aziridine ROCOP (improving upon the spontaneous pathways investigated so far). If successful, such an approach could allow the regular placement of urethane and amide linkages in the resulting polymers through terpolymerisation methods, which may deliver properties closer to the current industrial polyurethanes.

## 16 Conclusions and Outlook

Heterocycle/heteroallene ROCOP allows efficient and selective formation of many completely new and sophisticated polymer microstructures from comparatively simple monomers. Optimizing the catalysis should allow both the tailoring of the polymerization kinetics and delivery of useful materials for future applications to be prioritized. Although great improvements in carbon dioxide/epoxide ROCOP catalysis have been achieved, in many other cases the catalysis is really at an early stage. Key targets are increased reaction rates and control over tacticity, molar mass, linkage, and chain end reactions. One strategy is to seek inspiration from the demonstrated successful approaches in CO_2_/epoxide ROCOP catalysis. These include developing co‐catalyst‐tethered metal complexes/organocatalysts by exploiting heterodinuclear synergy and applying chiral or non‐initiating organometallic catalysts to properly control the stereo‐ or end‐group chemistry of the chain. There are, however, some monomer combinations for which optimized carbon dioxide ROCOP catalysts are unsuccessful and, in these cases, the field needs a better understanding of the kinetics and mechanisms so as to rationally improve performances. The polymerization catalysis community should feel optimistic in these endeavours, since the range and scope of ROCOP catalysts is still very narrow, with many Lewis acid and labile metal/organocatalysts remaining to be explored. Another priority is to develop tolerant catalysts and processes using impure monomers or mixtures—such systems would be highly attractive for large‐scale deployment but could also accelerate uptake by the broader polymer chemistry community.

In this Review, we have tried to highlight the immense potential for many under‐explored monomer combinations to deliver functionalised polymers and materials. The polymer chemistry and physics of these materials is at a very early stage and the field will benefit from the attention of those with expertise in processing, properties, and applications. One area worth immediate investigation is to use multi‐functional chain‐transfer agents to target new polymer architectures and topologies, for example explorations of star and brush polymers. Another is to exploit recently discovered switchable polymerization catalysis to deliver block‐sequence‐selective copolymers.^[241]^ The precise monomer placement within polymer chains afforded by switch catalysis has already shown promise in the delivery of ductile plastics, adhesives, and thermoplastic elastomers.[^60^, ^65^, ^242^, ^243^] In the future, its application with heteroatom‐functionalized polymers could be used to broaden into sectors including engineering plastics, fibre‐compatible resins, soft robotics, ionic conductors, and medical materials.

Ring‐opening copolymerization processes and polymers may be of interest to tackle UN Sustainable Development Goals (SDGs). Overall, polymer sustainability can only be assessed through life‐cycle assessments and are application‐specific, but there are some features of these polymers that meet criteria for sustainable polymers. For example, many monomers are existing industrial wastes and others could be bio‐derived. It is recommended that catalysis and polymer chemistry research should target such non‐petrochemical materials for development. The ROCOP process has high atom economy and may be suitable for retro‐fit into existing manufacturing and processing infrastructure. Polymer properties are well‐matched with growth industries in renewable energy generation, increased use of biomaterials such as wood/paper, or in delivering self‐repairing products. In terms of end‐life options, some of these polymers show promising characteristics for circular chemical recycling. These heteroatom‐containing backbones appear to facilitate depolymerisation to monomers or small cyclic molecules suitable for repolymerization under accessible conditions. Nonetheless, such properties must be carefully balanced with processing and application performances. Other heteroatom‐containing polymer scaffolds are biodegradable and are already finding application in medical sectors, which indicates that the by‐products of degradation are not toxic. Taken as a whole, such features highlight the potential of this interesting class of polymers in helping to tackle the problems of today's materials. Much more research is needed to improve their production, better understand their properties, and fully assess their life cycles.

## Conflict of interest

C.K.W. is a director of econic technologies

## Biographical Information

*Alex J. Plajer studied chemistry at the University of Heidelberg (BSc 2015) and the University of Cambridge (MPhil 2016). As a Cambridge Trust Vice Chancellor scholar, he studied the supramolecular realm of main group chemistry under the supervision of Prof. D. Wright and received his PhD in 2019. His current postdoctoral work under the supervision of Prof. C. Williams focusses on heterotrimetallic ROCOP catalysis and is supported by a Royal Commission for the Exhibition of 1851 research fellowship*.

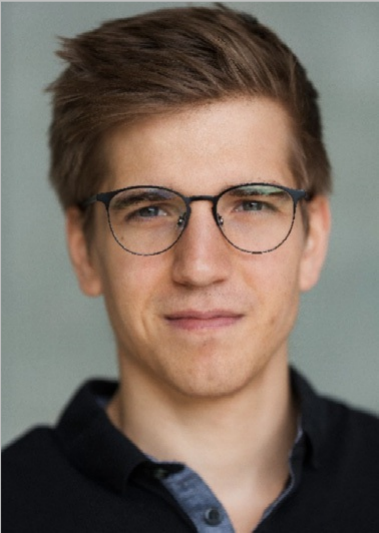

## Biographical Information

*Charlotte K. Williams obtained her BSc (1998) and PhD (2001; V. Gibson and N. Long) at Imperial College London. After postdoctoral research at the University of Minnesota (2001–2002, W. Tolman and M. Hillmyer) and Cambridge University (2002–2003, A. Holmes and R. Friend) she joined the Chemistry faculty at Imperial College London (2003–2016). She is currently a professor of Inorganic Chemistry at Oxford University. She recently became a fellow of the Royal Society (2021) and received an OBE for Services to Chemistry (2020), the Macro Group UK Medal (2019), and the DeChema Otto Roelen Catalysis Medal (2018)*.